# Sustaining Rare Marine Microorganisms: Macroorganisms As Repositories and Dispersal Agents of Microbial Diversity

**DOI:** 10.3389/fmicb.2017.00947

**Published:** 2017-05-29

**Authors:** Marc Troussellier, Arthur Escalas, Thierry Bouvier, David Mouillot

**Affiliations:** ^1^MARBEC, UMR IRD-CNRS-UM-IFREMER 9190, Université MontpellierMontpellier, France; ^2^Institute for Environmental Genomics, Department of Microbiology and Plant Biology, University of Oklahoma, NormanOK, United States; ^3^Australian Research Council Centre of Excellence for Coral Reef Studies, James Cook University, TownsvilleQLD, Australia

**Keywords:** microbial communities, microbial biodiversity, rare biosphere, microbiota, macroorganism–microbe interactions, dispersal, metacommunity, gut microbiota

## Abstract

Recent analyses revealed that most of the biodiversity observed in marine microbial communities is represented by organisms with low abundance but, nonetheless essential for ecosystem dynamics and processes across both temporal and spatial scales. Surprisingly, few studies have considered the effect of macroorganism–microbe interactions on the ecology and distribution dynamics of rare microbial taxa. In this review, we synthesize several lines of evidence that these relationships cannot be neglected any longer. First, we provide empirical support that the microbiota of macroorganisms represents a significant part of marine bacterial biodiversity and that host-microbe interactions benefit to certain microbial populations which are part of the rare biosphere (i.e., opportunistic copiotrophic organisms). Second, we reveal the major role that macroorganisms may have on the dispersal and the geographic distribution of microbes. Third, we introduce an innovative and integrated view of the interactions between microbes and macroorganisms, namely *sustaining the rares*, which suggests that macroorganisms favor the maintenance of marine microbial diversity and are involved in the regulation of its richness and dynamics. Finally, we show how this hypothesis complements existing theories in microbial ecology and offers new perspectives about the importance of macroorganisms for the microbial biosphere, particularly the rare members.

## Microbial Diversity in Marine Environments

The estimation of marine bacterial diversity has markedly increased in resolution during the last decade with the advent of high throughput sequencing technologies (Sunagawa et al., 2015; Louca et al., 2016). Although the total amount of microbial diversity is still a matter of debate, a total of one trillion (10^12^) species has been proposed (Locey and Lennon, 2015). The abundance distribution among so many species is particularly uneven with few dominant taxa and a very long tail of low abundant taxa, the later being undetectable using traditional clone sequencing and cultivation methods (Pedrós-Alió, 2012). Since the pioneer study of Sogin et al. (2006), an increasing number of studies show that the rare portion of communities constitutes most of the microbial diversity over large spatial and temporal scales (Szabo et al., 2007; Elshahed et al., 2008; Youssef and Elshahed, 2009; Vergin et al., 2013). This rarity feature of microbial communities has been reported in most, if not all, marine systems (Youssef et al., 2010; Campbell et al., 2011; Lynch and Neufeld, 2015) as observed for other microbial (e.g., in phytoplankton; Campbell et al., 2011; Hugoni et al., 2013; Lynch and Neufeld, 2015) or macroorganism communities (e.g., fish, plants or trees; Mouillot et al., 2013; ter Steege et al., 2013). Thus, rare microbes, those that have low abundance within communities, are more frequent than previously thought with widespread geographical distributions (Amend et al., 2013). Since rare microbes have only been characterized using cultivation-independent approaches, one cannot fully rule out whether rare operational taxonomic units (OTUs, an operational definition of a species based on 16S rRNA gene similarity) from different communities correspond to the same organisms or are the product of sequencing approaches limitations (Skopina et al., 2016). That being said, Galand et al. (2009) show that the rare members of microbial communities cannot reach a cosmopolitan distribution across the Arctic waters, but instead have a restricted geography suggesting that low-abundant taxa are also subjected to ecological constraints. In a study on the global distribution of 28,150 marine bacterial OTUs, Amend et al. (2013) revealed that spatially restricted OTUs (i.e., endemic) can be locally abundant in coastal ecosystems and thus that endemic microbes can be locally maintained without benefiting from source-sink dynamics from other habitats.

Generally, abundance and occurrence patterns observed for microbes differ from the classical abundance-occupancy relationship that prevails for macroorganisms, where locally abundant species tend to be geographically widespread while endemics tend to be locally rare (Gaston et al., 2000). Instead, the distribution of microbial diversity seems to be in agreement with the presence of a seed-bank of OTUs throughout the global ocean (Gibbons et al., 2013) and their repeated transitions from local rarity to prevalence driven by environmental variations, habitat heterogeneity, or stochastic events (Shade et al., 2014; Lynch and Neufeld, 2015). Although the ecological roles of these rare community members are still largely unknown, recent evidences suggest that they may benefit from local and short term environmental conditions that allows them to thrive and contribute to ecological key processes such as community resilience and stability (Alonso-Sáez et al., 2014; Aanderud et al., 2015; Shade and Gilbert, 2015). Since the ecological importance of rare microbial populations is unveiled, one of the main challenges is now to explain how some microbial taxa with low local abundance can have such widespread geographical distributions and how such a high marine microbial diversity can be maintained in local communities. In this quest, only some processes governing the ecology and dynamics of rare microbial taxa have been identified (Vergin et al., 2013; Lynch and Neufeld, 2015). However, to our knowledge, no studies have tried to reconcile these processes with existing theories in microbial ecology such as the Baas Becking’ *everything is everywhere* but *the environment selects* (Baas Becking, 1934) or the *killing the winner* theory (Thingstad and Lignell, 1997; Thingstad, 2000). More surprisingly, the influence of macroorganism–microbe interactions (McFall-Ngai et al., 2013; McFall-Ngai, 2015) on the ecology and dynamics of rare microbial taxa have been largely ignored despite the suggestion by some authors that macroorganisms may act as specialized habitats for rare microbial taxa in seawater (Frias-Lopez et al., 2002; Taylor et al., 2003; Sunagawa et al., 2010; Hao et al., 2015; Weiland-Brauer et al., 2015). Most of the literature dedicated to these interactions was focused on the importance of microbes for macroorganism’ nutrition, growth, survival, and well-being in general (for reviews see Nayak, 2010a,b; Clements et al., 2014; McFall-Ngai, 2015). The other side of the same coin, i.e., the importance of macroorganisms for microbe survival and abundance, has been mostly neglected. Hereafter, we will use the term “microbial communities” to refer to the complete set of bacterial and archaeal microorganisms present in a sample. The term “microbiota” will refer strictly to a host-associated microbial community, regardless of the type of host-microbe association. We define macroorganisms as multicellular eukaryotic organisms of size equal of bigger than meso-zooplankton organisms (i.e., >0.2 mm). Then, we will use the term “rare” and “abundant” to describe organisms that exhibit low and high abundance in local communities, respectively.

Here, we propose that macroorganisms contribute to the maintenance of microbial diversity at various scales in the marine environment. More precisely, we show how the influence of host-associated microbial communities can uphold rare environmental microbes in local communities, but also how macroorganisms have the potential to increase microbe dispersal and their spatial distribution. First, we review the results from the last decades on the main processes regulating marine microbial biodiversity. Second, we highlight that host-associated microbiota represent a significant part of marine microbial biodiversity. Third, we provide support that macroorganisms can constitute a favorable environment allowing the growth of rare marine microbial populations (i.e., rare opportunist copiotrophs) and act as a source of rare marine microbes. Fourth, we reveal the major role that some macroorganisms may have on microbial dispersal and their geographical distribution. Finally, we provide a new hypothesis describing the double role played by macroorganisms in the maintenance of microbial diversity through (i) their beneficial influence on rare community members and (ii) their role as dissemination vector for geographically restricted taxa. This new perspective, coined as “*sustaining the rare*,” is then discussed in the light of existing theories in microbial ecology.

## Review of the Main Processes Regulating Marine Microbial Diversity

The composition and structure of marine microbial communities are controlled by several factors acting simultaneously at different temporal and spatial scales (Fuhrman et al., 2008; Fierer and Lennon, 2011; Nogales et al., 2011). Hereafter, we review some of the main ecological processes, deterministic and stochastic, involved in these controls including bottom-up and top-down controls, micro- and macro scale heterogeneity, immigration and dispersal rates (Agogué et al., 2011; Stegen et al., 2015).

### Bottom-Up and Top-Down Controls

Bottom-up controls act through the availability of nutrients. One of the first mention of this type of control over microbial cells, if not the first, is the Baas Becking’ dictum “*everything is everywhere* but *the environment selects*” (Baas Becking, 1934). In its original paper, Baas Becking stated that latent microbial life could be resuscitated given the appropriate environmental conditions in selective culturing (de Wit and Bouvier, 2006). In marine pelagic ecosystems, microbial cells have to face two opposing sets of environmental conditions that would modify community composition and structure (Eilers et al., 2000; Jørgensen and Boetius, 2007). The first set can be referred as the “desert” environment, i.e., the large oligotrophic phase of marine pelagic ecosystems, where only the species able to use very low nutrient concentrations will maintain minimum growth, the others being rare or entering in dormancy. Dormancy, defined as “any rest period or reversible interruption of the phenotypic development of an organism” (Lennon and Jones, 2011), participates to the microbial seed bank along with other factors (e.g., sporulation, immigration; Pedros-Alio, 2006), and thus contributes to the maintenance of microbial diversity (Jones and Lennon, 2010). The seed bank has been defined as a reservoir of dormant cells and individuals that can potentially turn to an active state under future and more favorable environmental conditions (Mincer et al., 2007; Lennon and Jones, 2011). This concept differs somewhat from the notion of rare biosphere, which relates to the observation of few abundant species and a majority of rare ones in microbial communities (Newton and Shade, 2016). The hypothesis that both the seed bank and rare biosphere contribute to the maintenance of microbial diversity has been strongly supported by the recent finding that rare Archaea are able to become abundant at different time periods within the same habitat (Jones and Lennon, 2010) and by other results (Gibbons et al., 2013; Shade et al., 2014; Shade and Gilbert, 2015). The second set of environmental conditions is the “oasis” environment that offers higher nutrient concentrations, at least when compared with surrounding oligotrophic “deserts.” Highly nutritive environments like detritic organic particulate matter (Fuhrman, 1999; Kiørboe and Jackson, 2001; Kiørboe et al., 2002), zoo- and phytoplanktonic cells (Pinhassi et al., 2004; Grossart et al., 2005), macroorganism cadavers (Lebrato et al., 2011), sewage or oil pollution (Cappello et al., 2007) offer to some heterotrophic bacteria the opportunity to reach high growth rates (Hentschel et al., 2006; Smriga et al., 2010). Azam and Malfatti (2007) underline that organic matter in seawater is replete with transparent gels of different sizes, which provide huge surfaces for attachment and interaction of microbial cells. These authors propose that such gels promote niche or habitat diversity that contributes to the maintenance of the enormous genetic diversity of marine bacteria. To summarize, beyond the direct effect of nutrient patches, micro-heterogeneity offers bacteria a large range of micro-gradients and environmental niches that allow small populations to survive or even grow in specific micro-environments, even if these latter are only ephemeral.

Facing “desert” or “oasis” types of environmental conditions, two main groups of bacteria with distinct adaptive strategies were described (Poindexter, 1979, 1981). The first one corresponds to k-strategists, also described as oligotrophic organisms with an “equilibrium” type of growth strategy. Their metabolism is adapted environments with low-nutrient concentrations, such as the bulk water, where they exhibit slow and constant specific growth rates (<0.2 h^-1^). They cells tend to have a relatively small volume (<0.1 μm^3^), even between starvation and growth phases, which is often seen as a mechanism for avoidance of predation (Yooseph et al., 2010) and a mean to sustain a high surface-to-volume ratio that increases uptake efficiency of nutrients. k-strategists also tend to have a small genome (<2 Mbp) that contains few defense mechanisms and transcription regulators genes (σ-factors) while coding for a limited range of nutrient acquisition strategies (Lauro et al., 2009). The second group corresponds to r-strategists or copiotrophic organisms with a “feast and famine” type of growth strategy (Poindexter, 1981). These organisms can exhibit phases of rapid growth (>1 h^-1^) in nutrient-rich conditions but are generally outcompeted by k-strategists in nutrient-poor conditions (Giovannoni et al., 2014). r-strategists have a relatively large cell (>1 μm^3^) but exhibit size reduction in response to nutrient limitation. Their genome is large (2–4 Mbp) and code for many transcription regulators (including the *rpoS* involved in cell division regulation), defense (e.g., CRISPRs) and stress response mechanisms but also a variety of foraging related features (e.g., chemotaxis, motility, exoenzymes Lauro et al., 2009; Giovannoni et al., 2014). r-strategists organisms typically thrive in the “oasis” type of environments described above.

Top-down controls have been described as major forces shaping microbial communities and act through predation, that is grazing by other microorganisms (e.g., protists) and viral lysis (Kjelleberg et al., 1987; Suzuki, 1999). For instance, the “*killing the winner*” theory posits that in a given environment the more active microorganisms are the favorite targets of predators and phages (Thingstad and Lignell, 1997; Thingstad, 2000; Winter et al., 2010). These “winners” correspond to the best competitors for the available resources and are often the r-strategists able to exploit a resource patch and grow rapidly. Hence, microbial phages reduce the abundances of these “winners” and, doing so, avoid the exclusion of less competitive ones, preventing microbial communities to be (permanently) dominated by a limited number of taxa. Predation through grazing pressure may also shape the phenotypic and genotypic composition of bacterioplankton through the selection of grazing resistant bacteria (Jürgens and Matz, 2002; Sherr and Sherr, 2002; Troussellier et al., 2005; Alonso-Saez et al., 2009), and has been proposed as one of the driving forces of genome streamlining in oceanic bacteria as described in the “*cryptic escape hypothesis*” (Yooseph et al., 2010). This hypothesis suggests that success in limiting oceanic environment can be achieved by limiting biomass to discourage the adaptation of specific predators, i.e., by becoming ‘invisible’ to them as a food source (Yooseph et al., 2010). Additionally, even when grazing is limited and the available nutriments exert a strong selective pressure on microbial communities (e.g., oil), the microbial richness may remain high (Hernandez-Raquet et al., 2006). One of the reasons is that specialized microbial taxa, able to degrade complex molecules produce smaller “edible” molecules that can be used by a large number of taxa (co-metabolism process; Cottrell and Kirchman, 2000; Ciric et al., 2010).

Bottom-up and top-down controls are therefore major structuring forces of microbial communities that have shaped the above-described ecological strategies. In nature, both processes are acting simultaneously but their relative influence might differ according to the environment. For example, theory predicts that under “desert” type, low-nutrient conditions, competition will be the major factor shaping microbial communities so the best competitors for resources are predicted to be dominant (Holt et al., 1994; Leibold, 1996). This is the case in the more oligotrophic parts of the ocean where k-strategists specialized in the life at low nutrient concentration are the most abundant. On the contrary, in high-nutrient conditions (e.g., upwelling areas, coastal zones, highly productive high latitude waters, etc.), predation or top down-control is expected to be the major factor influencing microbial community structure. In that case, among the copiotrophs able to successfully forage on available nutrients, the ones that are the more able to avoid predation (i.e., defense specialists) are predicted to dominate (Bohannan and Lenski, 2000).

### Physical Transport and Large-scale Distribution

The dispersal of free-living microbes across the ocean can result from different processes acting at different scales. However, because of their very small size, microbial cells cannot disperse over large scale by their own means and they are passively transported from one place to another within water masses. Hence, as it is the case for other marine organisms, the dispersal capacity of bacteria depends on hydrodynamic conditions (Mincer et al., 2007; Hellweger et al., 2014). On the other hand, well-established seawater stratification may act as a physical barrier (clines, fronts). For instance, thermohaline ocean circulation generates distinct oceanic water masses, which tend to exhibit distinct specific prokaryotic communities (Agogué et al., 2011; Salazar et al., 2015; Sunagawa et al., 2015). Such large-scale segregation of water masses may limit dispersal and immigration of free-living microbes (Prairie et al., 2012). Along the vertical dimension, microorganism fluxes can be variable depending on the equilibrium between stratification and mixing forces (Mann and Lazier, 1991) but also depending on their association with sinking organic particles that may reach deep waters and bottom sediments (Simon et al., 2002). Upwelling phenomena also have the potential to influence the diversity of microbial communities, as demonstrated along the Brazilian coastal region (Cury et al., 2011).

### The Overlooked Role of Macroorganisms

Until recently, the role played by macroorganisms in the control of microbial diversity has been largely ignored in the literature (Mihaljevic, 2012; Saleem, 2015; Li et al., 2016); the interactions between hosts and their microbiota being mostly considered in the light of the potential effect, beneficial or deleterious, for the macroorganism’s life (Harris, 1993; Mao-Jones et al., 2010; Nayak, 2010b; Clements et al., 2014). Such a one-sided and asymmetric view of macroorganism–microbe interactions is exemplified by studies on fish and their intestinal microbial communities which generally focus on the benefits for the fish (Nayak, 2010a,b; Clements et al., 2014) or in a more limited way, how the fish gut microbiota support hosts’ functional roles (Smriga et al., 2010; Mouchet et al., 2012).

At the opposite side, two potential effects of macroorganisms on the diversity of seawater microbial communities need further attention. On one hand, an indirect effect, associated with the excretion and exudation of organic matter from macroorganisms, and which has been documented lately to stimulate bacterioplankton production (Fonte et al., 2011) but also its diversity (Vlahos et al., 2013) and abundance (Garren et al., 2008). On the other hand, a direct effect, through the introduction and dissemination of microbial organisms in the open seawater. Few studies have been published concerning the direct effect although potentially non-negligible as highlighted in this paper.

There is a clear distinction to make between the different types of macroorganism–microbe associations in order to evaluate their potential for the direct effect. Some host-associated microbes are specialists of particular environments in which they tend to be hyper abundant like for instance symbionts which live within the tissues of the host, but also mutualists and pathogens associated to digestive tract, skin and surface of macroorganisms (Pedrós-Alió, 2012; Bordenstein and Theis, 2015). These microbes depend on their relationship with the host and are expected to be part of the rare biosphere in free-living conditions encountered after being released by the host (actively or not) or after the death of the host (Pedrós-Alió, 2012). Another type of host-associated microbes are the opportunist copiotrophs which randomly encounter a macroorganism and are simply ‘passing by,’ benefiting from the rich nutrient conditions in the vicinity of their temporary host. These can be classified as commensalists and include for instance seawater or sediment microbes ingested during foraging and that can thrive in a macroorganism’s gut where nutrient or oxygen concentrations might be more favorable than in their original habitat (Smriga et al., 2010). In the following sections, we will show that marine macroorganisms host a large diversity of microorganisms and may constitute a nutrient-rich environment favorable to the growth of opportunistic microorganisms present at low abundance in seawater (Smriga et al., 2010). This nourishes the first part of the hypothesis developed here that marine macroorganisms, through the active or passive release of organisms from their microbiota, can be a source of diversified and active community members that seed the seawater rare biosphere (Pedrós-Alió, 2012; Vlahos et al., 2013).

## Macroorganisms Host Highly Diverse Microbial Communities

**FIGURE 1:**
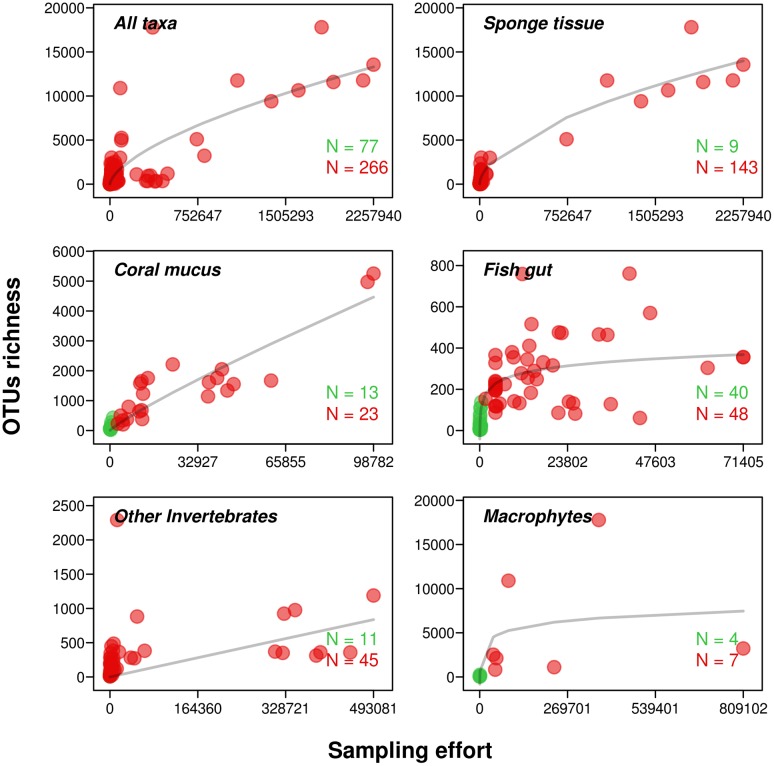
**Number of OTUs recorded in microbial communities associated with marine macroorganisms**. The sampling effort refers to the number of sequences obtained using clone-libraries (green) and pyrosequencing (red). OTU richness corresponds to the number of OTUs observed at 97% identity. Only one value of sampling effort and OTUs richness was retained per study and per species. When one species was sampled in different locations in one study, we used the mean richness and sampling effort. When only one location was sampled we used the total number of sequences and OTUs. Lines correspond to the best Species-Area Relationship model used to describe OTU-Sampling effort relationships.

The number of studies on the diversity of microbial communities associated with marine macroorganisms has increased exponentially during the last 5 years but there is surprisingly no synthesis considering various phyla. Here, we gathered information from the literature (82 studies) to highlight that these communities represent a significant part of the total marine microbial diversity. We collected sequence number (sampling effort) and OTU richness data obtained using sequencing approaches (i.e., clone-libraries and next-generation sequencing, NGS) and according to the following criteria: (i) at least one value of sampling effort and richness was reported per species and per study, (ii) a 97% similarity cut-off was used for OTU definition and (iii) availability of the total number of sequences and total richness or of the mean across replicates (more details are provided in **Appendix** and **Supplementary Table S2**). **Figure 1** represents the relationships between OTU richness and sampling effort in the microbiota of three groups of macroorganisms for which we found the more data (sponges, corals, and fishes) along with invertebrates (crustaceans, tunicates, molluscs, and echinoderms) and macrophytes for which sample size was lower. These data were fitted using “species area relationships” models (*mmSar* package in R; Guilhaumon et al., 2010) to determine the influence of increasing sampling effort on OTU richness (see Supplementary Material for details).

As a comparison, the estimated global richness in seawater microbial communities lies between ∼20,000 and ∼9,000 OTUs for coastal and open ocean surface waters, respectively (ICoMM data; Zinger et al., 2011). Higher estimates have been provided recently from the Tara Oceans expedition but including deep-water communities (Sunagawa et al., 2015).

The highest richness values in marine host-associated microbiota were observed for sponges (≈17,800 OTUs; Reveillaud et al., 2014) thanks to a sampling effort exceeding 2M sequences for some of the studied species. Recently, Thomas et al. (2016) showed that several sponge species taken separately can host as many OTUs as the surrounding seawater. When these authors combined their 804 samples representing 81 sponge species, the richness estimates reach up to 40,000 OTUs. Other studies, although less comprehensive, have previously reported that sponge microbiota and seawater microbial communities exhibit comparable richness values (Webster et al., 2010; Jackson et al., 2012).

The sampling effort for coral mucus microbiota was lower than for sponges (<100,000 sequences) but these communities still exhibit microbial OTU richness higher than the surrounding seawater (Sunagawa et al., 2010) and sediment (Carlos et al., 2013; Hernandez-Zulueta et al., 2016) for a given sequencing effort. The species-area (here sampling effort) relationships for sponge and coral microbiota were better fitted with a power model (*R*^2^ = 0.93 in sponge and *R*^2^ = 0.83 in coral), suggesting that the total diversity of microbes associated with these taxa is not asymptotic so has yet to be discovered.

The maximum richness value for marine fish gut microbiota was ≈800 OTUs (Givens et al., 2015) and the relationship with sampling effort was better described by a Monod model (*R*^2^ = 0.58) with an asymptote at 372 OTUs. Although it suggests that increasing the sampling effort may not significantly increase the richness value of fish gut microbial communities, the number of sampled fish species was limited, especially for high sampling effort, and this result is highly influenced by few extreme observations.

Other marine host-associated microbiota were less studied (e.g., crustaceans, tunicates, molluscs, and echinoderms) and we combined the associated data. The overall OTU richness was lower than for other studied taxa, for instance Gao et al. (2014) detected more than 2,000 OTUs in the gut of a sea cucumber (Echinoderm). When observations for all these groups were analyzed together, the OTU-sampling effort relationship was better described using a logistic model (*R*^2^ = 0.35) with an asymptote at 611 OTUs.

The microbiota of other vertebrates than fish have been characterized only recently and tend to show high richness, probably due to multiple body parts being sampled simultaneously (e.g., mouth, skin, rectum, stomach, and shell). For instance a richness of more than 30,000 OTUs was observed for sea turtle (*Chelonia mydas*; Price, 2016), between 5,247 (Bik et al., 2016) and 11,465 OTUs (Soverini et al., 2016) for dolphin (*Tursiops truncatus*), and around 965 OTUs for the sea lion (*Zalophus californianus*; Bik et al., 2016).

Finally, the microbiota OTU richness associated with marine macrophytes (i.e., seagrasses and macroalgae) appears to be quite high, although our estimation is limited to 11 samples, and tends to be higher than the surrounding seawater (Mancuso et al., 2016). For instance, values as high as 17,779 OTUs were observed in epiphytic communities of *Corallina officinalis* (Brodie et al., 2016). The best model describing the OTU-sampling effort relationship was a Monod model (*R*^2^ = 0.35) with an asymptote at 9,251 OTUs.

When considering all taxa together, the OTU-sampling effort relationship was better described by a non-asymptotic power model (*R*^2^ = 0.66), confirming that microbial diversity has not yet been fully explored in marine host-associated microbiota. However, collected data come from studies using different technologies and bioinformatic tools while various mechanisms are involved in the assembly of host-associated microbial communities. These two limitations prevent direct comparisons across taxa in our approach. Nonetheless, collected data support the conclusion that these communities represent a non-negligible part of the total richness of marine microbes.

## Macroorganisms as Sources of Rare Taxa for Seawater Microbial Communities

Several studies have shown that the composition of seawater and sediment microbial communities may greatly differ from those associated to macroorganisms (sponges: Dupont et al., 2013; Thomas et al., 2016; corals: Sunagawa et al., 2010; Carlos et al., 2013; fishes: Balcázar et al., 2010; Schmidt et al., 2015; Parris et al., 2016; molluscs: Pfister et al., 2014; macrophytes: Burke et al., 2011; Aires et al., 2013; Mancuso et al., 2016; marine mammals: Bik et al., 2016). Therefore, marine macroorganisms constitute a unique and large variety of niches for environmental microbes and host a significant part of the overall marine microbial diversity. The crucial question is whether the rare OTUs of environmental microbial communities are also found in low abundance within host-associated microbiota or whether macroorganisms offer a best-suited environment for the rare biosphere. To address this, we observed the frequency distribution of OTUs in microbial communities associated with different macroorganisms and in the surrounding seawater, focusing on the changes in relative abundance for rare microbial taxa (<1%). Here we differentiated two types of rare environmental microbes, the symbionts/mutualists whose natural habitats is the host microbiota and the opportunist copiotrophs which thrive in commensal associations with macroorganisms.

### Host-associated Microbiota As Hotspots of Rare or Undetected Microbial Taxa in Open Seawater

**FIGURE 2:**
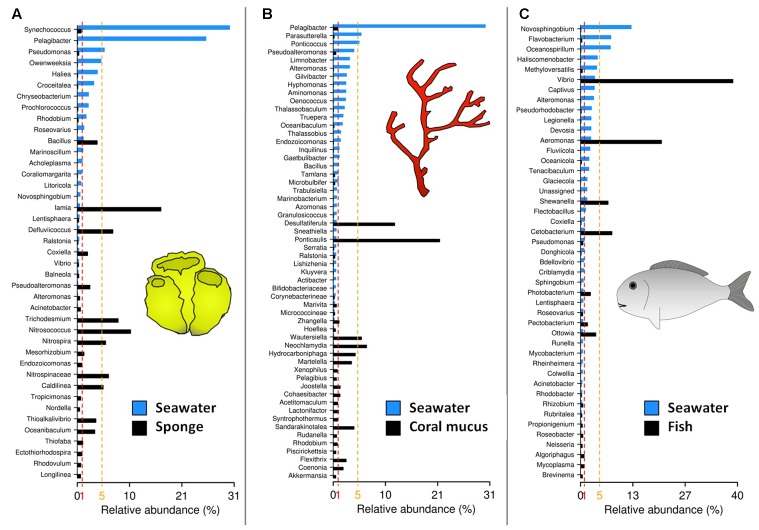
**Abundance distribution of OTUs within host-associated microbiota and the surrounding seawater microbial communities**. Data from **(A)** Webster et al. (2010); **(B)** Fernando et al. (2014); **(C)** Schmidt et al. (2015).

A good example of the dynamics between environmental microbes and macroorganisms comes from the squid (*Euprymna scolopes*), which periodically releases its fluorescent symbiotic bacteria *Vibrio fisñheri* into seawater from which it can transit to infect newborn squids (Lee and Ruby, 1994). Taylor et al. (2012) tested the hypothesis that “sponge specific” OTUs are not specific but rather rare enough in other environments so they cannot be detected by conventional molecular methods. Using 12 million reads of 16S rRNA genes from 649 seawater, sediment, hydrothermal vent and coral samples, they show that a significant number of so-called “sponge specific” OTUs (77/173, i.e., 45%) are in fact present but rare in other marine environments. Besides, these microbes commonly associated with sponges, show contrasted abundances between sponge microbiota and other habitats of marine ecosystems. One of the most striking examples is the study from Webster et al. (2010; **Figure 2A**) where bacterial genera such as *Iamia*, *Nitrosococcus*, or *Trichodesmium* shift from less than 0.8% relative abundance in open water to 16.9, 10.8, and 8.3% of the total abundance in sponge microbiota, respectively. These examples are only a small part of the studies reporting such an OTUs abundance shift between seawater or sediment and the sponge microbiota (Lee et al., 2011; Jackson et al., 2012; Cuvelier et al., 2014; O’Connor-Sánchez et al., 2014; Polónia et al., 2014; Reveillaud et al., 2014; Cleary et al., 2015; de Voogd et al., 2015; see Supplementary Figures for more examples).

Coral microbiota also appear to host high relative abundances of rare OTUs found in seawater communities (**Figure 2B**). For instance, Carlos et al. (2013) show that most of genera associated with coral mucus are heterotrophic aerobes, which are commonly found in a wide range of hosts, suggesting that the coral mucus is a suitable habitat for opportunist organisms. They observe that *Sphingomonas* sp., *Pseudomonas* sp. along with some Gammaproteobacteria show relative abundances lower than 1% in seawater and 1.6% in sediment but up to 3.2, 6.5, and 12.3% in the mucus of coral species. In the same vein, Fernando et al. (2014) report that dominant OTUs in coral microbiota are rare in seawater and vice versa. Here again, this type of abundance shift has been observed in several studies (Hentschel et al., 2006; Sunagawa et al., 2010; Hernandez-Zulueta et al., 2016).

A similar trend, although involving different microorganisms, have been observed for epiphytic microbial communities of marine macrophytes such as the green alga *Ulva australis* (Burke et al., 2011), *Caulerpa racemosa* (Aires et al., 2013), and the kelp *Macrocystis pyrifera*. Mancuso et al. (2016) show that microbial families such as Saprospiraceae and Phyllobacteriaceae can shift from 0.01% abundance in seawater to more than 5% in the microbiota of the brown algae *Cystoseira compressa*.

To our knowledge, few studies have compared NGS results from marine fish microbiota and surrounding seawater. For instance, Schmidt et al. (2015) report relative abundance shift between fish microbiota and seawater for four genera, i.e., *Vibrio*, *Aeromonas*, *Cetobacterium*, and *Shewanella* (**Figure 2C**).

This first set of data showing that rare seawater bacteria may be found in quite higher abundance within host-associated microbiota correspond to symbiotic and mutualistic relationships (Schmitt et al., 2012; Taylor et al., 2012) or epibionts (Aires et al., 2013; Mancuso et al., 2016). These OTUs can be in turn a source for the seawater rare biosphere either through active or passive expulsion from their host (Jones et al., 2007), or when this latter dies. Epibionts can be removed by renewal of host surface through mucus secretion, shedding of the cuticula or epidermis and many other mechanisms (e.g., Wahl, 1989).

### Macroorganism Digestive Tracts Increase the Relative Abundance of Transient and Rare Seawater OTUs

The digestive tracts of different marine animals are shown to make favorable habitats for transient and rare seawater bacteria. These gut environments are particularly important for our hypothesis since they tend to be associated with motile organisms which increase the dispersion potential of the microbes they host (see Macroorganisms as Dissemination Vectors for Marine Microbes). For example, Aronson et al. (2016) characterize the gut microbiota of a deep-sea snail (*Rubyspira osteovora*) and reveal that the taxa dominating gut communities (*Mycoplasma, Psychromonas*, and *Psychrobacter*) are either not detected or with a very low abundance in the surrounding seawater environment. In the same way, Zhang et al. (2016) observe that dominant taxa from the gut communities of the chinese mitten crab (*Eriocheir sinensis*), all affiliated to the Mycoplasmataceae, are almost absent from seawater communities. Parris et al. (2016), performed the only NGS-based comparison dealing strictly with fish gut and seawater microbial communities. Their results show that most of the OTUs forming the core of fish gut microbiota (Damselfish and Cardinalfish) are either absent or present at very low abundances in surrounding seawater, suggesting that a selective accumulation of these taxa occurs in fish guts. A similar trend is observed in the transient gut microbial communities of seahorses (*Hippocampus guttulatus*) where the dominant OTUs differ from the surrounding seawater (Balcázar et al., 2010). Regarding the microbiota of marine mammals, Bik et al. (2016) report that the 10 most abundant OTUs associated with dolphin rectum account for 81% of the reads while their proportion in surrounding seawater is only of 0.05%. The gut of marine Copepods has also been shown to favor the growth of rare seawater microbes, with notably several groups of Gammaproteobacteria (*Pseudoalteromonas, Glaciecola* spp., Halomonadaceae, and *Marinobacter* spp.) or Firmicutes (Planococcus spp.) shifting from very small abundance in seawater to dominance in the Copepod guts (Moisander et al., 2015). This shifting in relative abundance was observed in other macroorganisms such as Tunicate (Erwin et al., 2011, 2014) and Copepods (Shoemaker and Moisander, 2014) but also on the skin microbial communities of amphibian (Walke et al., 2014), which is a topic in itself. Overall, an interesting feature is that seawater microbial communities, notably in the open ocean, tend to be dominated by organisms that are characterized by an oligotrophic life style such as *Pelagibacter* or *Prochlorococcus*, while host-associated microbiota are often dominated by taxa reported as copiotrophs, such as *Alteromonadaceae* and *Vibrionaceae* or *Bacteroidetes* (Lauro et al., 2009; Teira et al., 2009; Yooseph et al., 2010; Vergin et al., 2013; Spring et al., 2015).

To summarize, this data synthesis supports the hypothesis that macroorganisms constitute a favorable environment for the growth of rare seawater microbes whether the nature of the association with the host is symbiotic, mutualistic or simply opportunist.

### Marine Animals’ Microbiota Increase Absolute Microbial Abundance

Fish gut microbial communities are among the most studied microbiota and there is a consensus that microbial abundances are higher than in the surrounding water. Collected data from the literature on colony forming units (CFU) and total counts (TC) within the gut content of 27 marine fish species (**Supplementary Table S3**) show that TC range from 1.1 × 10^8^ cells.mL^-1^ (*Coryphaenoides yaquinae*; Yano et al., 1995) to 2.6 × 10^11^ cells.mL^-1^ (*Epinephelus coioides*; Sun et al., 2009). Nearly half of the reported TC data are >1.0 × 10^10^ cells.mL^-1^. These values are between 2 and 4 orders of magnitude (100 to 10,000) higher than the maximum TC values reported for seawater microbial communities (between 5.0 × 10^5^ and 10^6^ cells⋅mL^-1^; Gram et al., 2010). Sponges are described as microbial incubators providing suitable conditions for rapid growth and high abundances (10^9^ cells.cm^-3^ of sponge tissue; Webster and Thomas, 2016) while enhanced microbial activity can be detected in the feces of coral reef fish (Smriga et al., 2010) and zooplankton (Tang et al., 2010), with evidence that this boosted activity can last up to 2 days (Hargrave, 1976). Faster microbial growth rates leading to higher absolute abundance, compared to seawater, is reported within the microbiota of polychaetes, holothurids, deep-sea amphipods (Harris, 1993), sponges (Hentschel et al., 2006) and zooplankton (Tang et al., 2010). In a synthesis on gut microbial communities of aquatic invertebrates, Harris (1993) highlights that such increases in microbe numbers are not necessarily beneficial to the host, which may be viewed as an incubator providing more suitable conditions for microbial growth in terms of organic substrates compared to surrounding seawater.

To conclude, it appears that the various types of associations with macroorganisms are particularly favorable to rare environmental microbes by simultaneously increasing their relative and absolute abundances in host-associated microbiota.

## Macroorganisms as Dissemination Vectors for Marine Microbes

By definition, microbial plankton dispersal depends on the movement of water masses. The proportion of non-motile cells in the marine environment is estimated between 40 and 80% of the total biosphere (Stocker, 2012). Even in the motile fraction of marine microbes, motility is an intermittent and energetically costly process that is mainly considered as a support for chemotaxis responses to patchy resources at small spatial scales (Mitchell et al., 1995; Barbara and Mitchell, 2003). Hence, motility is not sustainable over long time periods and cannot be sufficient to allow microbial dispersal over large distances. Sessile macroorganisms discussed in the previous sections such as macrophytes, sponges, corals, ascidians and some molluscs are, by definition, not expected to provide assistance to microbe dispersal.

**FIGURE 3:**
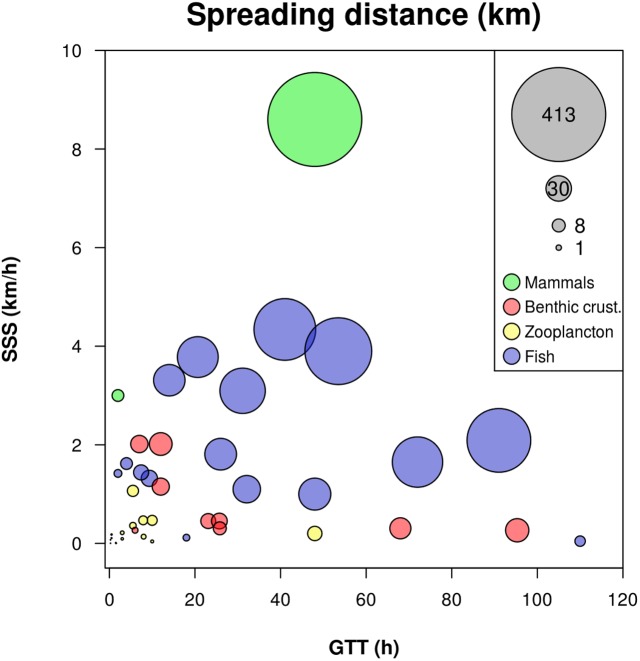
**Spreading distance in different groups of marine macroorganisms**. Spreading distance (SD in km) is estimated as the product of gut transit time (GTT in h) and sustainable swimming speed (SSS in km.h^-1^). The surface of the bubbles is proportional to SD. Data correspond to **Supplementary Table S1**.

On the contrary, mobile marine animals can exhibit high dispersal abilities and can thus contribute to the transportation of associated microbial communities over long distances. In this section we focus on transient gut microbial communities since the skin microbiota of marine animals are still largely overlooked (but see Chiarello et al., 2015). Horizontal and vertical transport of gut bacteria associated with mobile marine animals can be controlled by at least three intrinsic characteristics: (i) gut transit time (GTT), (ii) ability to cross a given horizontal or vertical distance in a given time (swimming speed, SS) and (iii) ability to move from a pelagic to a benthic habitat and vice-versa. The product of GTT by SS defines the SD, i.e., the estimated distance over which gut bacteria can be transported by their host. We collected data from the literature to evaluate the dispersion potential of gut microbes by estimating the SD for several groups of marine macroorganisms. We used information at the species level or provided a range at the smallest possible taxonomic unit depending on the data (**Supplementary Table S1** and **Figure 3**).

The maximum SD estimated for benthic invertebrates such as crabs and shrimps is around 25 km. These unexpectedly high values arise from long GTT in crabs (>95 h) and relatively fast SS for shrimps (∼2 km.h^-1^). As a group, decapods exhibited a mean SD of 14.3 ± 8.1 km. Gammarids can only support SD lower than 10 km. These estimates are derived from combinations of disconnected studies and the real dispersion potential depends on GTT and swimming behavior, which are influenced by temperature and foraging behavior for the former aspect and by currents and habitat structure for the latter. Although data to estimate the SD of organisms living in the sediment (e.g., Nereis) are not available, these organisms are known for participating actively in bioturbation and consequently are involved in microbe dispersal within the sediment.

The GTT estimated in zooplanktonic organisms such as Copepods an Euphausids ranges from 0.26 to 3.00 h and from 1.5 to 10 h, respectively (Atkinson et al., 1996; Perissinotto and Pakhomov, 1996; Irigoien, 1998), while nominal SS ranges between 3 and 93 m.h^-1^ (Yamazaki and Squires, 1996). Thus, SD of gut microbes is ≤300 m for Copepods and ≤1 km for Euphausids. Mysids are bigger and tend to have both longer GTT and faster SS so their SD can reach 5.9 km. In addition, zooplankton realizes daily vertical migration allowing the vertical transport of free-living bacteria across density gradients, which would be otherwise impenetrable for them (Grossart et al., 2010). This dispersal potential appears higher than the one that microbial cells can reach by their own means, but still relatively small compared to other macroorganisms.

In this regard, fishes are a more efficient vector of dispersal for their gut microbes. GTT values among the 16 species for which we gathered information appear quite variable, with an average of 36.3 ± 31.8 h and extreme values of 2 and 110 h. These GTT are in accordance with Grosell et al. (2011) who reported values ranging from 10 to 158 h for marine carnivorous fishes. For fishes, we will consider sustained swimming speed (SSS), which is a good indicator of prolonged swimming abilities allowing fishes to cross significant distances (Videler and Wardle, 1991). As observed for GTT, the collected SSS values were highly variable (1 to 100 km.d^-1^) and covered the range of possible SSS, as even the largest pelagic fishes (e.g., tunas or marlins) cannot exceed 100 km.d^-1^ (Block et al., 1992). For the 16 species considered here, SD ranges between 2 and 190 km, corresponding to a dispersal potential from 200 to 200,000 times greater than the one microbial cells can reach by themselves (considering microbes swim between 15 and 100 μm.s^-1^; Wadhams and Armitage, 2004). Furthermore, mesopelagic fishes perform daily vertical migrations between the epipelagic layer where they forage and deeper layers where they digest. In the Bering Sea, the transport of material from surface to deeper layers is estimated at 15,000 tons.day^-1^ (Radchenko, 2007), which is low compared with marine snow flux but which is achieved over shorter time periods. Such back and forth movements across habitats (pelagic-benthic, surface-deep layer), may allow marine microbes to cross chemo- or thermoclines which normally represent geographic dispersal barriers (Schaus and Vanni, 2000; Brenner and Krumme, 2007). One-step further, fish predators may convey microbial communities over larger distances through predation and transfer them across food webs. For instance, marine mammals preying on fishes exhibit SSS equivalent or higher than the fastest fishes (i.e., 72 to 206 km.day^-1^; Watanabe et al., 2011). Their intestinal length is correlated with body size and ranges from 11 m in sea otters to 100 m in fin whales (Williams et al., 2001). Although the average GTT of most marine mammals is relatively short (2 h to 2–3 days) considering their intestine length (Carter et al., 1999), their SD can be higher than 400 km. Thus, marine mammal predators exhibit a very high potential for the transport of their own gut microbes but also those of their prey.

In conclusion, pelagic microbes that are ingested and travel inside macroorganisms’ guts are “extracted” from the seawater matrix where viscosity makes a strong constraint to their mobility and dispersal. Whatever the horizontal and vertical dispersion potential of motile macroorganisms, even a quick transit through their guts allow planktonic microbes to make “giant steps” compared to their own capacity. This highly efficient way of dispersal could benefit to free-living microbes in several manners, for instance to locate new resources, escape unfavorable conditions, colonize new patches or avoid competition. This process has also the potential to seed local communities with organisms from another place, thus increasing the spatial distribution of rare microbes. Considering their dispersal potential and their gut transit time, macroorganisms are more likely to be involved in spatial dynamics at local (e.g., between coral reefs or habitats within a lagoon) or meso scales (e.g., between coastal lagoons or islands) than at large scale where dispersal across water masses depends more on sea currents (Amend et al., 2013; Giovannoni and Nemergut, 2014).

## Sustaining the Rare

### Rationale and Hypotheses

**FIGURE 4:**
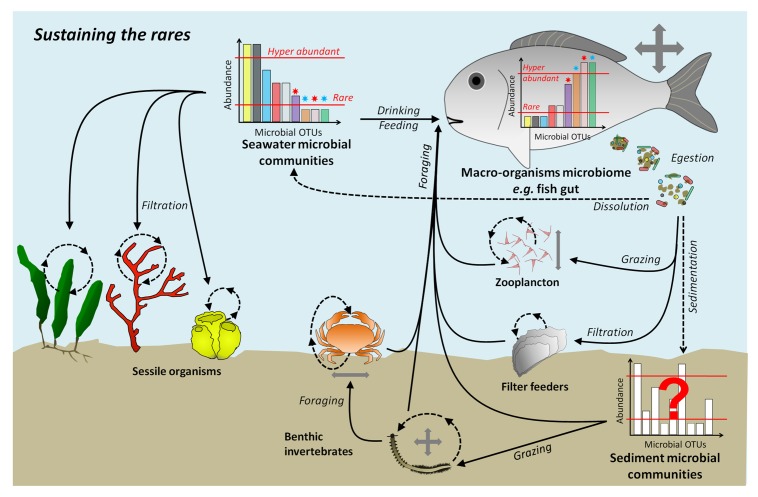
**Sustaining the rare: schematic view of macroorganisms, and especially fishes, as key contributors of microbial diversity maintenance**. As highlighted in the main text, macroorganisms are expected to participate in the maintenance of marine microbial diversity by favoring certain rare taxa in seawater and sediment microbial communities but also through their horizontal and vertical transfer capacities. Full arrows depict transfer of microbes through trophic processes and dashed arrows depict microbial release from macroorganisms and passive processes. Gray arrows correspond to macroorganisms’ horizontal and/or vertical transfer potential. Asterisks on the abundance distributions represent the two types of host-associated microbes discussed in the main text, symbionts/mutualists (blue) and opportunist copiotrophs (red). Interrogation points depict ecosystem compartments for which there is currently not enough available data to generalize the proposed mechanisms of macroorganisms impact on microbial diversity.

When taken together, empirical evidences support the argument that macroorganisms play a so far overlooked role in the dynamics and diversity of seawater microbial communities across scales. More precisely, the core idea of this review is that aquatic macroorganisms “*sustain the rare taxa*” or in other words participate to the maintenance of rare environmental taxa (**Figure 4**). To support our hypothesis, we firstly highlighted some features of marine microbial diversity: (i) most of diversity is represented by low abundance taxa (i.e., rare), which constitute a seed-bank, and (ii) even locally rare taxa are present over large spatial scales. Another feature is that microbial rank-abundance curves are highly dynamic with rare taxa from the microbial seed-bank becoming dominant under particular environmental conditions, habitat heterogeneity, and stochastic events (Lynch and Neufeld, 2015; Shade and Gilbert, 2015). Second, we show that macroorganisms, whether in their tissue, their mucus or in their gut, host a non-negligible part of marine microbial diversity and consequently should be considered as a key microbial habitat in the same way as water and sediment. Third, we accumulate evidences that host-associated microbiota represent a source of rare microbial taxa found in seawater. This process encompasses two types of microorganisms. On one side the non-planktonic microbes (symbionts, mutualists, and pathogens) whose ecology is dependent on the host and that, consequently, can only be observed at low abundance once released in the environment. On the other side, the opportunist copiotrophs (r-strategists) that can benefit from nutrient rich environments provided by their temporary host (gut, mucus, or skin) to increase their abundance and activity before returning to the environment where their abundance will decrease until they contribute to the rare biosphere. Fourth, we show that the high dispersion potential of some marine animals, makes them a dissemination vector able to reseed large areas with abundant and highly active bacteria (Smriga et al., 2010). Recently, Shade et al. (2014) reported synchronous occurrence patterns of some conditionally rare taxa (CRT) and suggested common environmental drivers or shared sources of dispersal for these synchronous CRT. Such synchronized patterns could be generated by dispersal of naturally co-occurring organisms through host-associated microbiota, followed by simultaneous release in a given location where their enhanced activity might allow them to thrive temporarily. All the above results concur with the observation that even rare organisms are widely distributed in the marine environment (Gibbons et al., 2013; Lynch and Neufeld, 2015) and that marine macroorganisms might directly participate to this homogenization at local or meso scale (1–100 km); large scale geographic distributions being under the influence of global currents and circulation (Mincer et al., 2007; Cury et al., 2011; Giovannoni and Nemergut, 2014).

### Link with Other Theories

**FIGURE 5:**
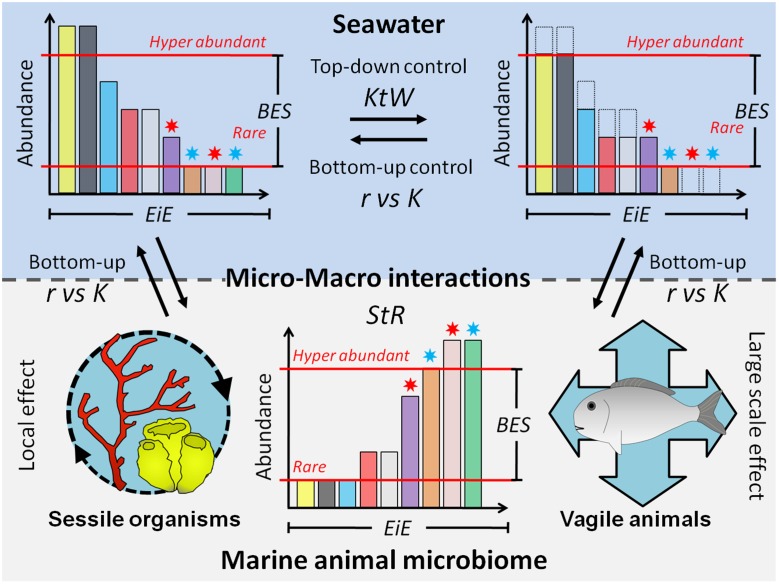
**Relation between “sustaining the rare” hypothesis and other classical microbial theories**. StR: Sustaining the Rare, the hypothesis introduced in this paper. EiE: Everything is Everywhere – BES: But Environment Selects, the two tenets of Baas Becking dictum. KtW: Killing the Winner, top-down control of the more abundant and fast growing microbial population through viral lysis. r vs. K: ecological strategies of microbes influencing their response to bottom-up and top-down factors of control. Asterisks on the abundance distributions represent the two types of host-associated microbes discussed in the main text, symbionts/mutualists (blue) and opportunist copiotrophs (red).

In our view, the concept of “*sustaining the rare*” (StR) and the associated hypotheses presented above constitute more than a conceptual view of the interactions between micro and macroorganisms. Indeed, the underlying principles of StR make it complementary, and not mutually exclusive, with other theories in microbial ecology such as the Baas Becking’ “*Everything is everywhere* but *the environment selects*” (EiE-BES) and the Thingstad’ “*Killing the winner*” (KtW; **Figure 5**).

The most well-known theory in microbial ecology is the classic Baas Becking’ dictum “*Everything is everywhere* but *the environment selects.*” This corresponds to the idea that while all microbial life is distributed worldwide, most of the microbial taxa are only latently present in a given environment (Baas Becking, 1934). Baas Becking himself recognized that considering that “*everything is everywhere*” requires that microbial cells are transported and distributed homogeneously over the globe (de Wit and Bouvier, 2006). Here is the first link with our StR hypothesis. Indeed, we provide evidence that motile macroorganisms constitute a dissemination vector for some environmental microbes, enhancing their dispersal to homogenize microbial communities at large scale. In a previous analysis of the original Baas Becking’ paper, de Wit and Bouvier (2006) mention that despite the development of novel culture-independent approaches it would be impossible to obtain evidence for the “*everything is everywhere*” tenet. However, a recent study comparing a deeply sequenced water sample (∼10 M 16S rRNA reads) with 356 ICoMM datasets show the existence of a persistent microbial seed-bank throughout the global ocean (Gibbons et al., 2013), which is clearly in accordance with the EiE dictum. Here, we advocate that marine megafauna may play a significant, although rarely considered, role in the large-scale homogenization of seawater microbial communities. Again, it is important to differentiate between symbionts/mutualists, which, by definition, are restricted to the environments where their host is present and the opportunist copiotrophs, which are the ones expected to be upheld by macroorganisms. Concerning the second part of Baas Becking’ dictum, “but *the environment selects*,” it is now acknowledged that the consequences of environmental selection correspond to changes in the relative abundance of community members rather than changes in community composition *per se* (i.e., presence–absence; Gibbons et al., 2013; Shade et al., 2014). Here is the second linkage between Baas Becking’ dictum and *sustaining the rares*, with marine animal microbiota acting as environments in which selection operates and thus expected to favor temporarily rare seawater microbes. In that case too, the association with the host can provide different benefits to symbionts/mutualists (e.g., protection against predation, provision of particular nutrients) and to opportunist copiotrophs (e.g., enhanced biomass and activity).

Another well accepted theory in microbial ecology is “*killing the winner*” (Thingstad, 2000), which refers to the selective predation by phages and grazers on the superior competitors within a community, allowing the weaker competitors to increase in density. Hence, *killing the winner* is expected to favor the coexistence of several taxa within a community, avoiding the hyper-dominance by just a few taxa and preventing microbes from immobilizing all the limiting nutrients in microbial biomass. *Killing the winner* is based on the existence of trade-off between competition and defense specialists, the former being expected to dominate the community in resource depleted environments while the latter being favored in nutrient rich environments (Winter et al., 2010). Hence, *killing the winner* and *sustaining the rare* can be seen as mirror theories, with the former preventing the dominance of competition specialists, while the latter favor the maintenance of defense specialists (i.e., the opportunist copiotrophs) by providing them with an environment where they can thrive but also through the release of external mucus and fecal pellets that can serve as nutrient patches.

As described in *sustaining the rare* hypothesis, host-associated microbiota represent local communities that are connected together by dispersal and constitute a metacommunity at a larger scale (Leibold et al., 2004). The maintenance of regional species diversity in a metacommunity depends on the presence of different environmental conditions within local communities, along with asynchronous fluctuations and dispersal of organisms between them (Leibold et al., 2004; Venail et al., 2008). In such context, when environmental conditions change in a community and are not favorable anymore to the species present, dispersal ensures that species adapted to these new conditions are available to replace less adapted ones. In theoretical models, this allows maintenance of ecosystem processes at large scale and reduces their variation in variable environmental conditions (Loreau et al., 2003; Hector et al., 2010; Shanafelt et al., 2015); it has been coined as the spatial component of the insurance hypothesis (Loreau et al., 2003; Wang and Loreau, 2014). If we replace the view presented in this article into the metacommunity framework, macroorganisms represent various environmental conditions for microbes and play an important role in microbial dispersal, suggesting they may participate to the spatial insurance (resistance and resilience) of the microbial processes at the metacommunity level (Baho et al., 2012; Mihaljevic, 2012). Additionally, theoretical models predict that changes in connectivity between communities following perturbation may substantially alter both species diversity and ecosystem processes at local and regional spatial scales (Gonzalez and Chaneton, 2002; Mouquet et al., 2003). In such scenario, the observed biodiversity erosion of macroorganisms in marine ecosystems (i.e., defaunation; Estes et al., 2011; Mora et al., 2011; McCauley et al., 2015) may ultimately impact on the spatio-temporal dynamics of microbial metacommunities and the ecosystem processes they sustain. These considerations need further attention notably as it was recently stated that “*nothing is known about how the aquatic ecosystem trophic downgrading is linked to the microbiome diversity loss and associated ecosystem services*” (Saleem, 2015).

## Conclusion and Future Directions

Several processes have been proposed to explain the maintenance of the rare biosphere in marine microbial communities, or the large geographic distribution of organisms with small dispersal abilities. Surprisingly, the influence of macroorganisms on microbial diversity and dynamics has been greatly overlooked. Here, we gathered evidences that the microbiota of marine macroorganisms can now be considered as a key part of the microbial diversity within the marine environment. Further, we present new hypothesis, coined as “*sustaining the rare*,” which integrates the interactions between microbes and macroorganisms into a metacommunity framework, to highlight the importance of macroorganisms in the maintenance of microbial diversity and the spatio-temporal dynamics of the rare biosphere. To conclude, our analysis of the literature supports our hypothesis, but further testing and experimental validation through hypotheses-driven experiments are needed. One simple way to test this hypothesis experimentally is to compare the dynamics of the same initial seawater microbial community incubated in controlled mesocosms with and without macroorganisms, but also with various levels of macroorganism diversity. One can expect limited changes in the diversity of microbial communities in mesocosms without macroorganisms, while mesocosms with one or a combination of macroorganisms are expected to show both an increase in microbial richness and a change in dominant OTUs by enrichment from macroorganisms. For instance, one study tested experimentally the effect of a co-culture of shrimp, crab, and shellfish on seawater microbial communities in a mariculture pond (Li et al., 2016). Their results suggest that macroorganisms induced a change in the dominant OTUs of bacterial communities since they observed differences between the ponds with and without invertebrates. This topic is only in its infancy but given the current defaunation of all oceanic systems we urge scientists to investigate more closely the potential key role of macroorganisms’ microbiota in sustaining marine microbial diversity, particularly the rare taxa.

## Author Contributions

MT initiated the study. MT and AE wrote the manuscript. TB and DM contributed substantially to manuscript revisions. MT and AE collected the data from literature. AE analyzed the data and made the figures.

## Conflict of Interest Statement

The authors declare that the research was conducted in the absence of any commercial or financial relationships that could be construed as a potential conflict of interest.

## Supplementary Material

APPENDIX S1 **Methodology and additional results**. Description of data gathering methodology. Presentation of additional results. List of references presented in Supplementary Tables S1, S2, S3.

TABLE S1 **Dispersion potential of gut microbial communities by different groups of macroorganisms**. Gut transit time (GTT), sustained swimming speed (SSS) and the corresponding spreading distance (*SD* = GTT × SSS) for different marine macroorganisms. For Fishbase reference, when several data are reported, the mean was used. For non-fish groups we provide only minimal and maximal ranges.

TABLE S2 **Number of OTUs recorded in microbial communities associated with different groups of marine macroorganisms**. Data were reported for sequencing-based methods, i.e., clone-libraries and NGS.

TABLE S3 **Abundance and culturability within fish gut microbiota**. Total cell counts were estimated using epifluorescence microscopy and culturable cell counts using culture-dependent methods. The proportion of culturable microbial cells is also reported when data allow its calculation.

## Abbreviations

GTT: gut transit time time required for food to transit through the digestive track
OTU: operational taxonomic unit an operational definition of a species based on 16S gene similarity
SD: spreading distance the distance over which gut bacteria can be transported by their host estimated as GTT × SS(S)
SS: swimming speed
SSS: sustainable swimming speed indicator of prolonged swimming abilities allowing aquatic organisms to cross significant distances.
