# Supplementary material for: Sustaining Rare Marine Microorganisms: Macroorganisms As Repositories and Dispersal Agents of Microbial Diversity
Source: Front Microbiol. 2017 May 29;8:947. doi: 10.3389/fmicb.2017.00947 (PMC5447324; doi:10.3389/fmicb.2017.00947)
Supplement: TABLE S3 — Abundance and culturability within fish gut microbiota. Total cell counts were estimated using epifluorescence microscopy and culturable cell counts using culture-dependent methods. The proportion of culturable microbial cells is also reported when data allow its calculation. [file Presentation_1.PDF]

Supplementary material

1- Data gathering methodology

1.1 – Sampling effort and OTUs richness data

The quantitative data presented in Figure 1 and table S1 were recovered using the following rules:

- we chose to present one value of sampling effort and richness per species and per study.
- we used only OTUs richness obtained with a 97% similarity cut-off as this is the most frequently used threshold, thus increasing the amount of available data.
- we used the values reported by the original authors when provided in the main text, in tables of in supplementary information. When only rarefaction curves were available, we used a plot digitalization approach (http://arohatgi.info/WebPlotDigitizer/app/).
- when no data was directly available we contacted the original authors.
- when available, we used the total number of sequences and OTUs detected for each species analyzed in the original paper.
- when the total numbers were not available, we used the the mean richness and sampling effort values estimated across the different location where the species was sampled or across the different replicates analyzed.

1.2 – Estimation of sampling effort - OTUs richness relationship

To estimate the relationship between the sampling effort and the OTUs richness in macro-organisms associated bacterial communities, we used the *mmSAR* package (Guilhaumon *et al.* 2010) to fit species area relationships (SAR). We tested the six following models: power, exponential, monod, logistic, ratio and lomolino. The best model was determined using the Akaike information criteria (AIC).

1.3 – Abundance shifts

The quantitative data presented in Figure 2 and supplementary figures S2-S6 were recovered from the VAMPS database (Huse *et al.* 2014) or from supplementary material of original papers. Figure 2 present data grouped at the genus level. The figures S2-S6 exhibit various level of taxonomic resolution.

1.4 – Spreading distance

The quantitative data presented in Figure 3 were recovered from original papers. When both GTT and SSS were available for one given species we combined them. When a range was presented we used the two extremes values. When no data were available at the species level we use ranges of data collected from various studies on a given group.

2- Additional results

Table S1 – Results of model comparison.

	All taxa		Sponge tissue		Coral mucus		Fish gut		Other invertebrates		Macrophytes	
	AIC	R2	AIC	R2	AIC	R2	AIC	R2	AIC	R2	AIC	R2
power	4898.35	0.66	2000.36	0.93	452.47	0.83	843.14	0.55	650.82	0.28	191.35	0.29
expo	5161.04	0.26	2296.47	0.48	489.92	0.53	839.26	0.57	648.31	0.31	191.23	0.29
monod	4928.65	0.63	2042.32	0.90	453.49	0.83	837.18	0.58	672.06	-0.06	190.38	0.35
logist	4912.44	0.65	2021.42	0.92	452.07	0.84	839.67	0.58	646.89	0.35	190.62	0.44
ratio	4930.66	0.63	2044.32	0.90	455.04	0.83	839.19	0.58	648.97	0.33	192.38	0.35

Figure S1 – Number of OTUs recorded in bacterial communities associated marine macro-organisms.

Same as figure 1 in the main text but for small sampling effort values. The first figure represents 0.2% of the sampling effort and the second represents 0.05%

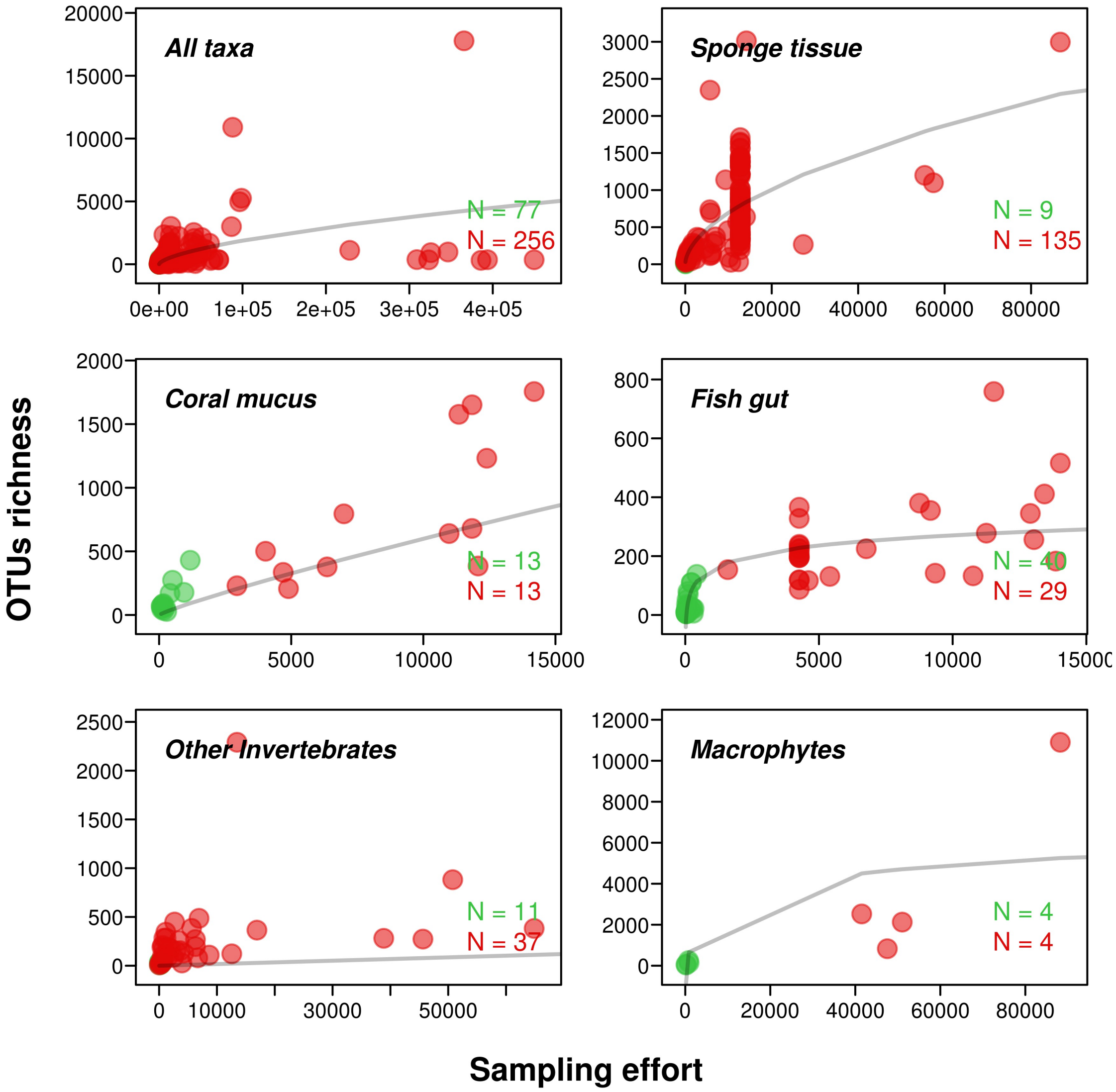

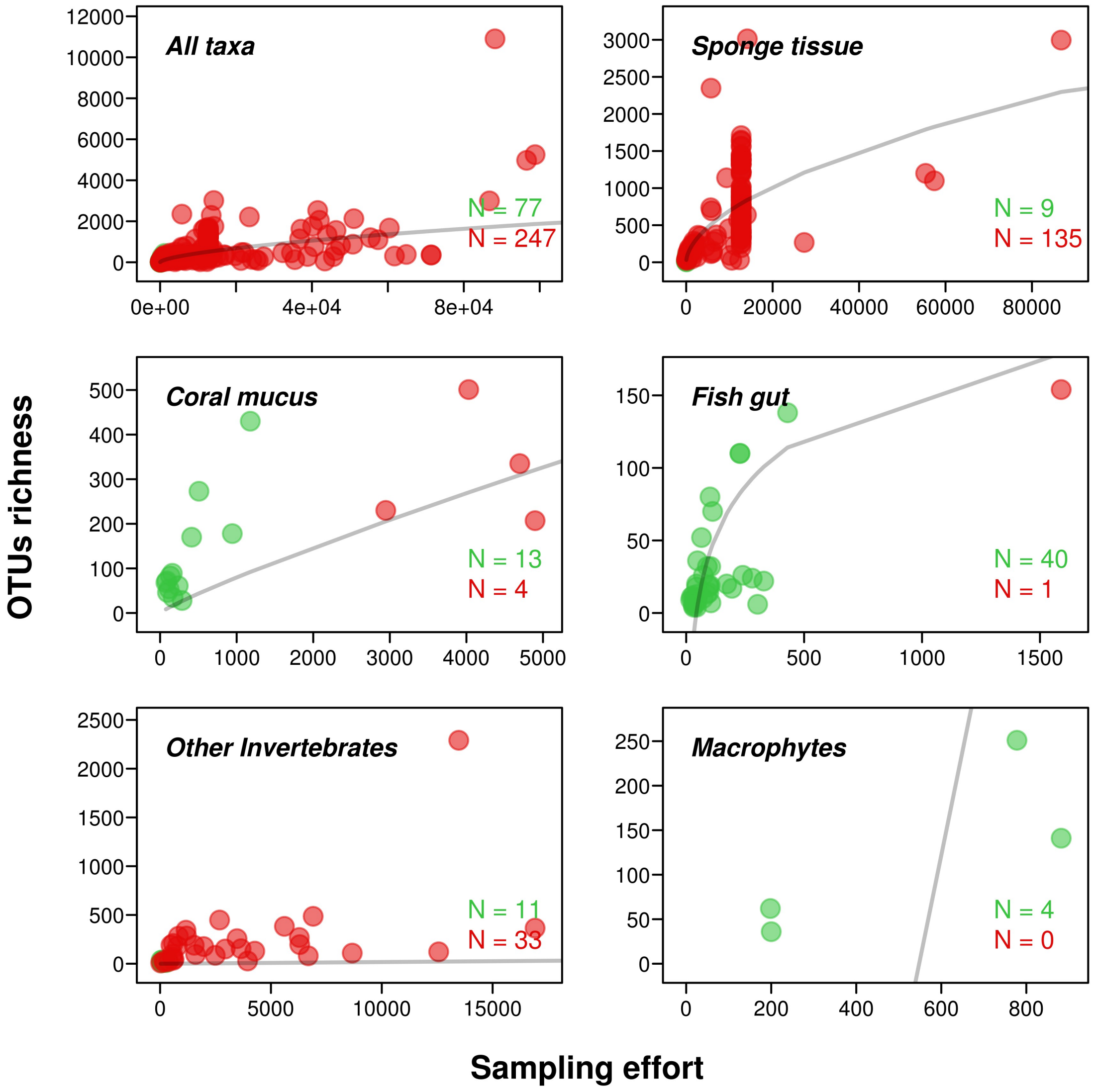
All taxa
N = 77
N = 247
Sponge tissue
N = 9
N = 135
Coral mucus
N = 13
N = 4
Fish gut
N = 40
N = 1
Other Invertebrates
N = 11
N = 33
Macrophytes
N = 4
N = 0
OTUs richness
Sampling effort

Figure S2-S6 – Abundance distribution of OTUs within macro-organisms associated bacterial community and the surrounding seawater bacterial communities.

The following plots were constructed in the same way as the figure 2 in the main text. However, the retrieved data have a coarser level of resolution which varies depending on the study. Only the taxa with total abundance > 0.5% are represented.

Data from Balcazar *et al.* 2010

Hippocampus guttulatus

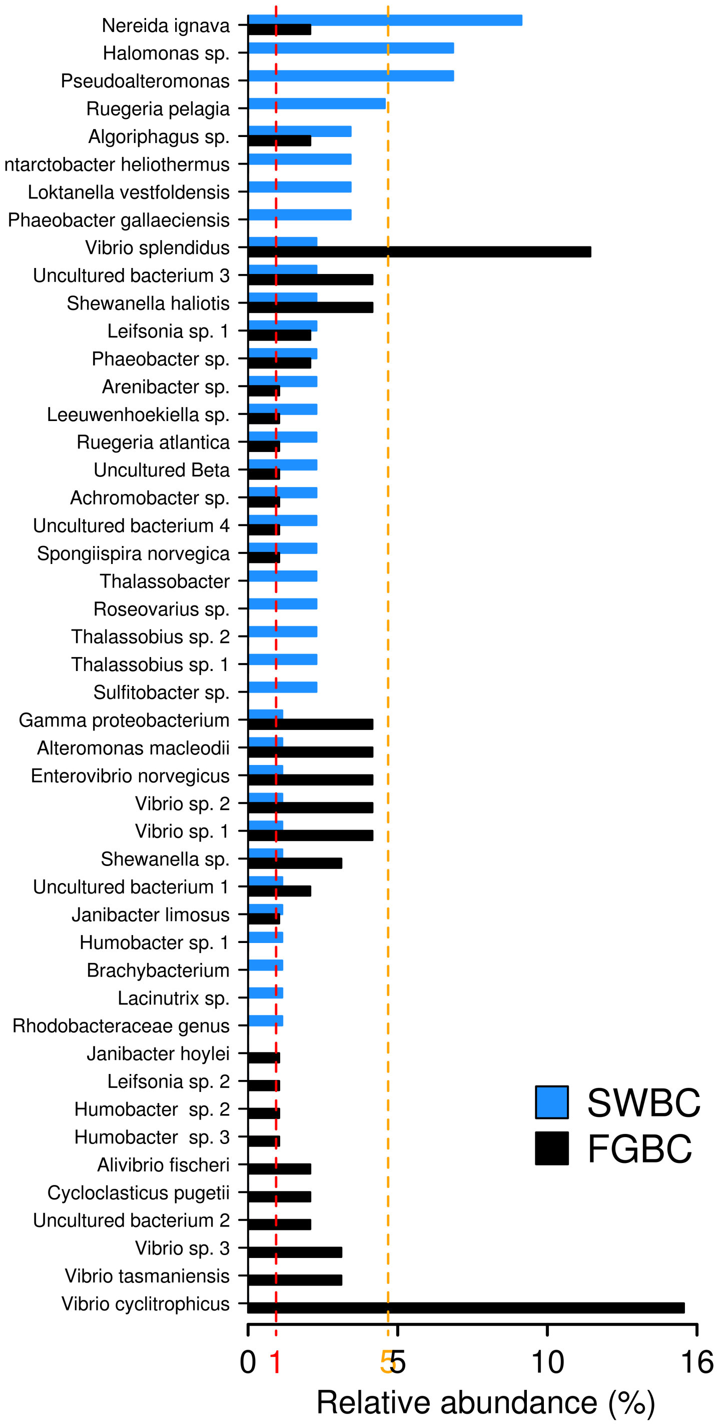

Data from Carlos *et al.* 2013

Madracis decactis, Mussismilia hispida, Palythoa caribaeorum, and Tubastraea coccinea

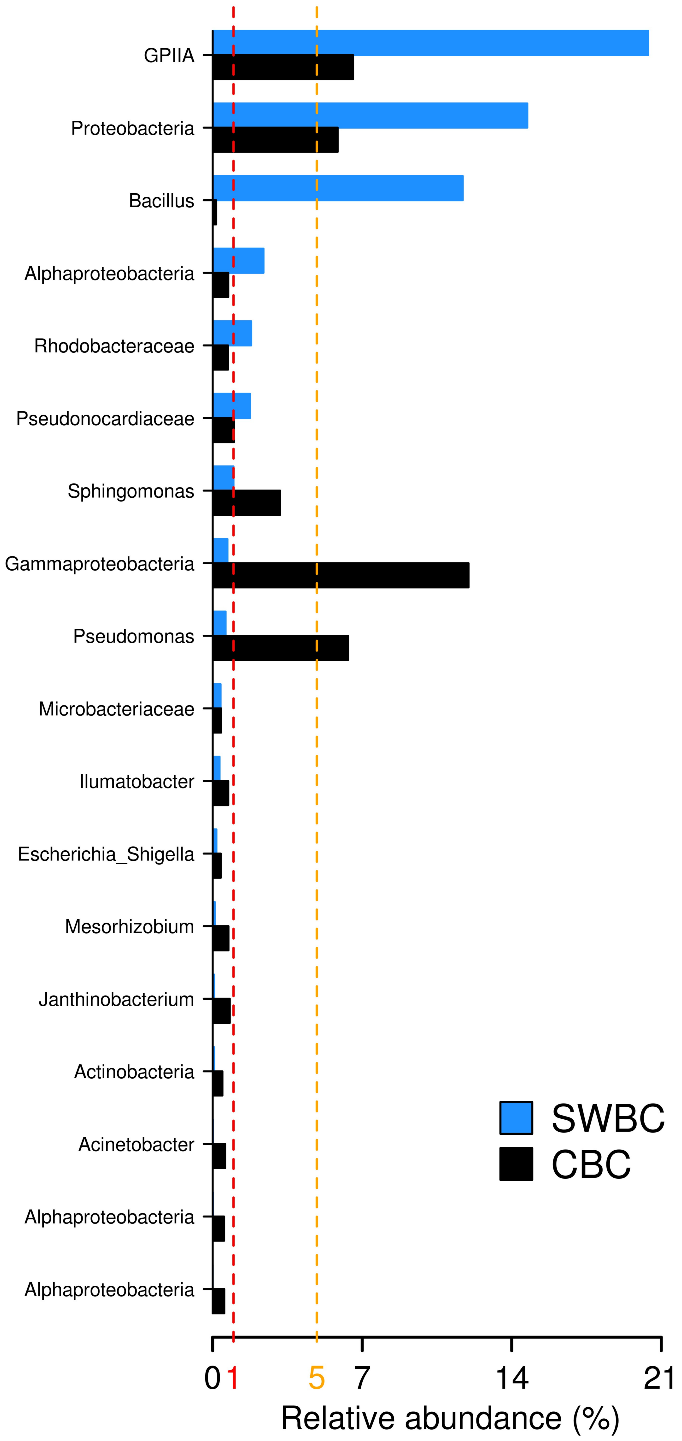

Data from de Voogd *et al.* 2015

Stylissa massa and Xestospongia testudinaria

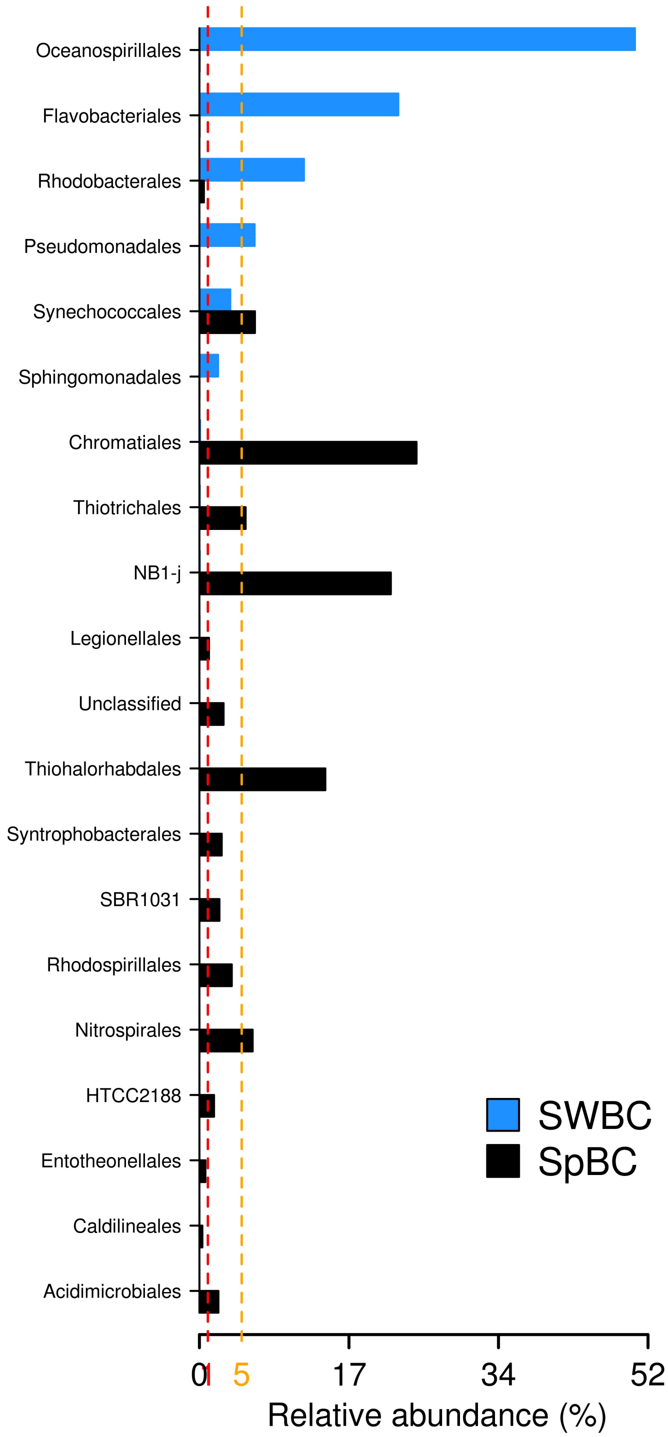

Data from Erwin *et al.* 2011

Didemnum sp.

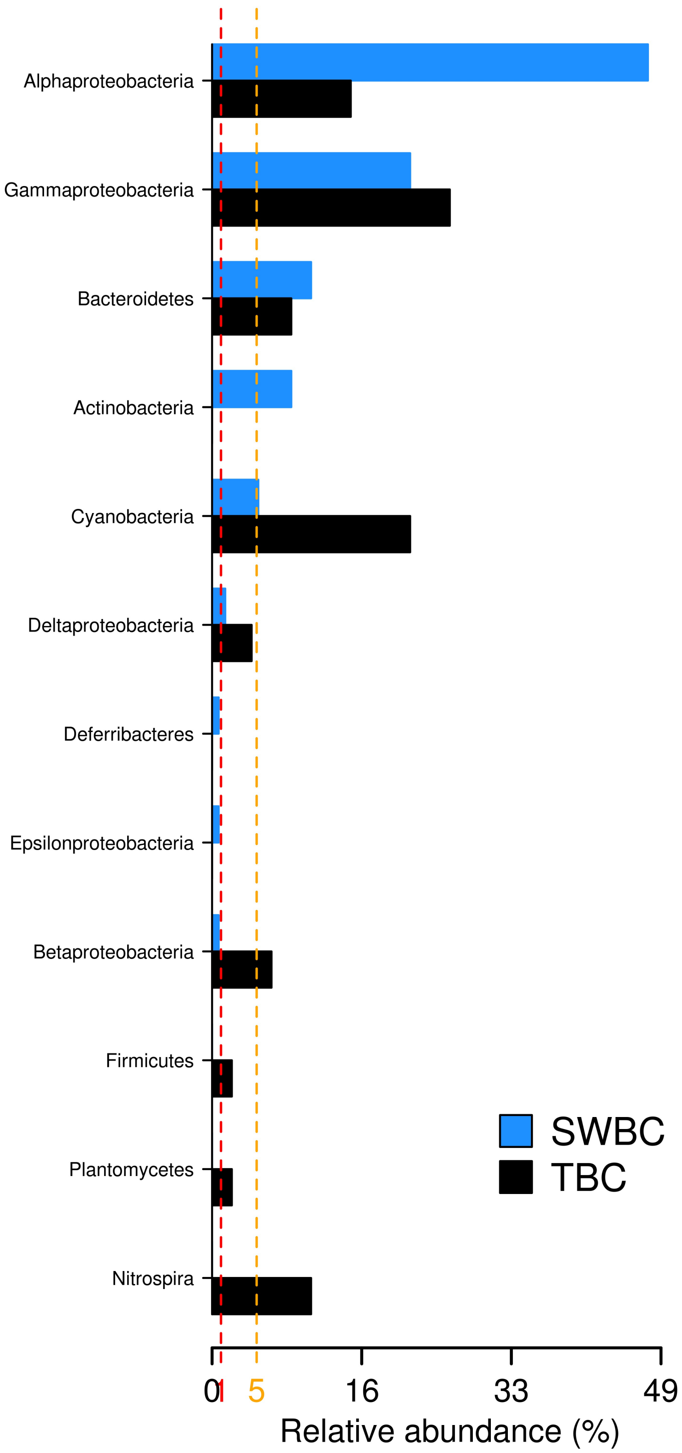

Data from Sunagawa *et al.* 2010 (VAMPS project: ICM_CCB_Bv6)

Montastraea spp., Diploria strigosa, Porites astreoides, Acropora spp. Gorgonia ventalina

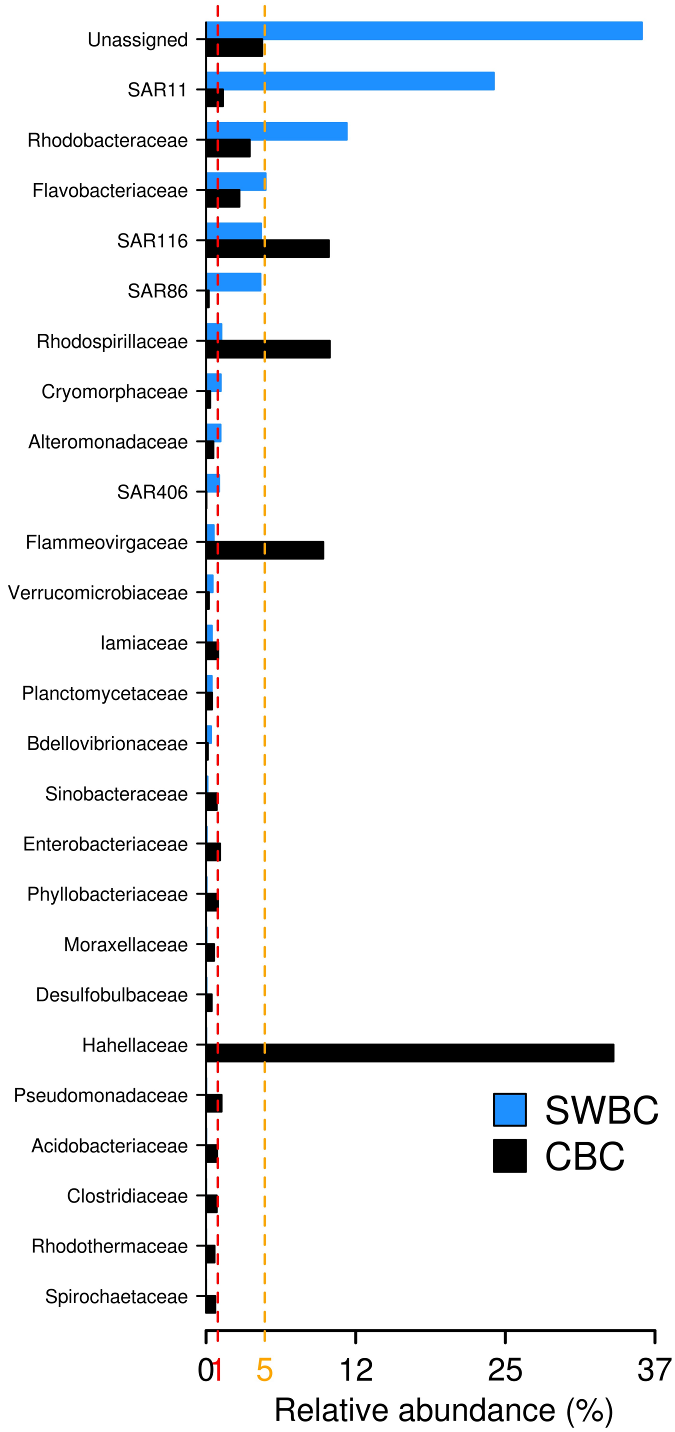

Data from Hentschel *et al.* 2006

Several species

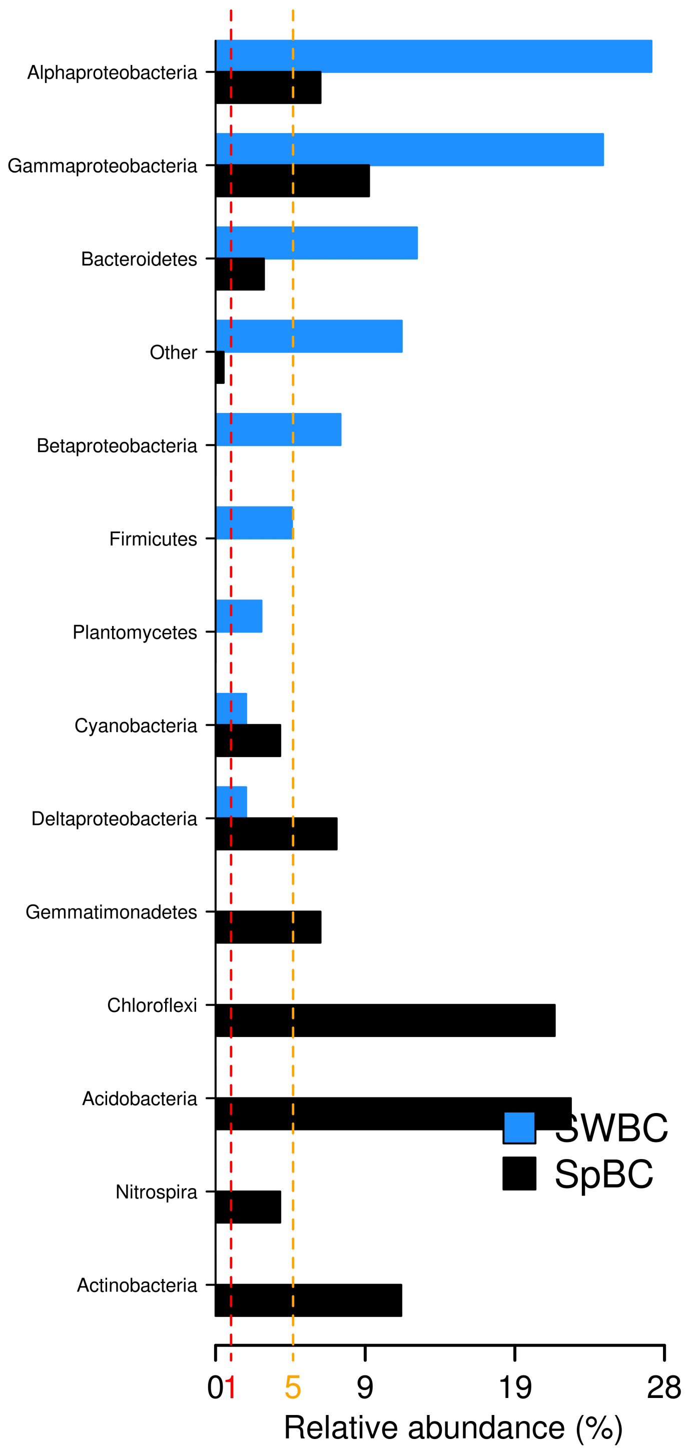

Data from Bik *et al.* 2016

Several mammals

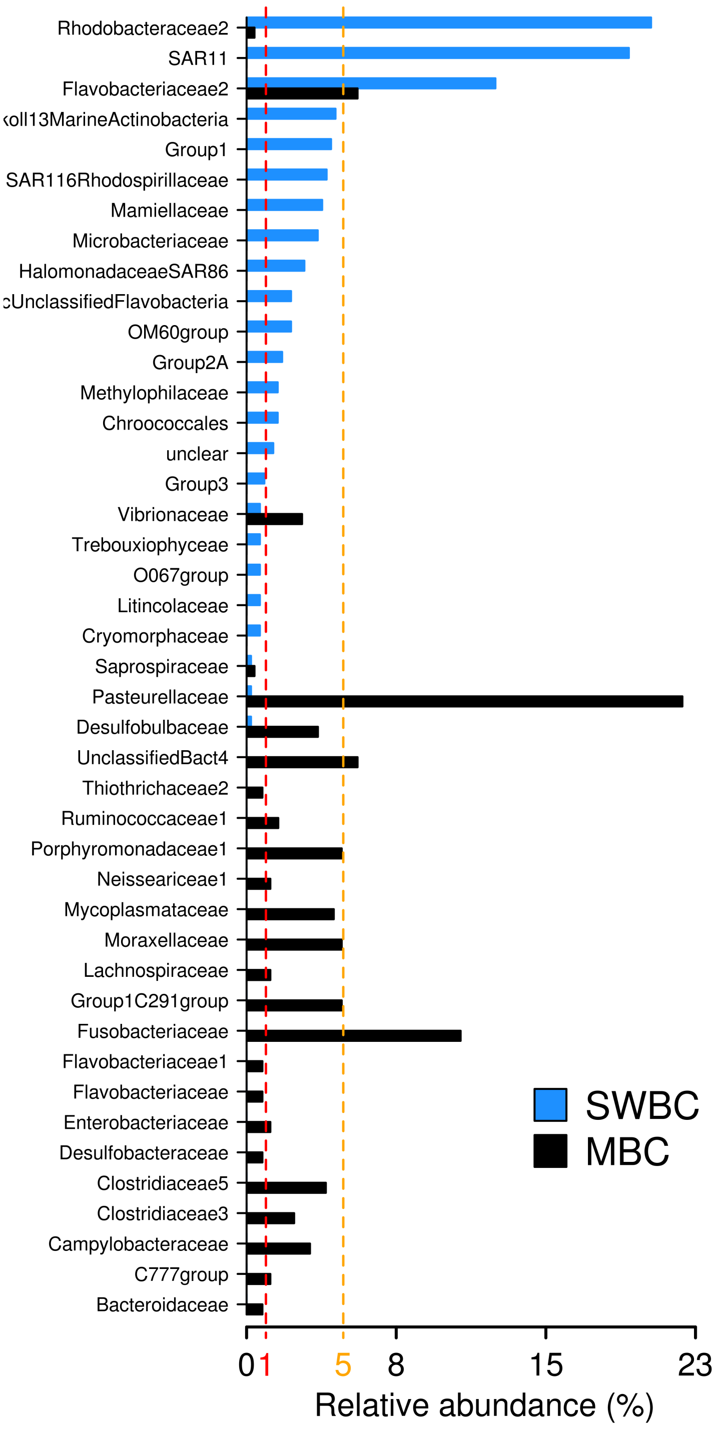

3- References used in tables

3.1 – References used in Table S1

ÁLVAREZ, A., GARCÍA, B.G., VALVERDE, J.C., GIMÉNEZ, F.A. & HERNÁNDEZ, M.D. (2009) Gastrointestinal evacuation time in gilthead seabream (Sparus aurata) according to the temperature. *Aquaculture Research*, 1101–1106.

ATKINSON, A., WARD, P. & MURPHY, E.J. (1996) Diel periodicity of Subantarctic copepods: relationships between vertical migration, gut fullness and gut evacuation rate. *Journal of Plankton Research* **18**, 1387–1405.

BAILEY, D.M., BAGLEY, P.M., JAMIESON, A.J., CROMARTY, A., COLLINS, M.A., TSELEPIDIS, A. & PRIEDE, I.G. (2005) Life in a warm deep sea: Routine activity and burst swimming performance of the shrimp Acanthephyra eximia in the abyssal Mediterranean. *Marine Biology* **146**, 1199–1206.

BASARAN, F., OZBILGIN, H. & OZBILGIN, Y.D. (2007) Comparison of the swimming performance of farmed and wild gilthead sea bream, Sparus aurata. *Aquaculture Research* **38**, 452–456.

BEAUDREAU, A.H. & ESSINGTON, T.E. (2009) Development of a new field-based approach for estimating consumption rates of fishes and comparison with a bioenergetics model for lingcod (Ophiodon elongatus). *Canadian Journal of Fisheries and Aquatic Sciences* **66**, 565–578.

BENKWITT, C., BRODEUR, R., HURST, T. & DALY, E. (2009) Diel feeding chronology, gastric evacuation, and daily food consumption of juvenile Chinook salmon in Oregon coastal waters. *Transactions of the American Fisheries Society* **138**, 111–120.

BOOTH, M. A., TUCKER, B.J., ALLAN, G.L. & FIELDER, D.S. (2008) Effect of feeding regime and fish size on weight gain, feed intake and gastric evacuation in juvenile Australian snapper Pagrus auratus. *Aquaculture* **282**, 104–110.

BROWN, R. & GEIST, D. (2002) Determination of swimming speeds and energetic demands of upriver migrating fall chinook salmon (Oncorhynchus tshawytscha) in the Klickitat River,. Washington.

CARTER, S.K., ROSAS, F.C.W., COOPER, A.B. & CORDEIRO-DUARTE, A. (1999) Consumption rate , food preferences and transit time of captive giant otters Pteronura brasiliensis : Implications for the study of wild populations. *Aquatic Mammals* **25**, 79–90.

CLAIREAUX, G., COUTURIER, C. & GROISON, A.-L. (2006) Effect of temperature on maximum swimming speed and cost of transport in juvenile European sea bass (Dicentrarchus labrax). *The Journal of experimental biology* **209**, 3420–3428.

COWLES, D.L. & CHILDRESS, J.J. (1988) Swimming Speed and Oxygen Consumption in the Bathypelagic Mysid Gnathophausia ingens. *October*, 111–121.

DARCY, G.H. (1985) Synopsis of biological data on the pinfish, Lagodon rhomboides (Pisces: Sparidae).

EGLI, D. & BABCOCK, R. (2004) Ultrasonic tracking reveals multiple behavioural modes of snapper (Pagrus auratus) in a temperate no-take marine reserve. *ICES Journal of Marine Science* **61**, 1137–1143.

FERNANDEZ, M., IRIBARNE, O.O. & ARMSTRONG, D.A. (1994) Swimming behavior of Dungeness crab, Cancer magister Dana, megalopae in still and moving water. *Estuaries* **17**, 271–275.

FIALHO, C., BANHA, F. & ANASTÁCIO, P.M. (2016) Factors determining active dispersal capacity of adult Chinese mitten crab Eriocheir sinensis (Decapoda, Varunidae). *Hydrobiologia* **767**, 321–331.

FROESE, R. & PAULY, D. (2000) FishBase 2000: concepts, design and data sources.

HANDELAND, S.O., IMSLAND, A.K. & STEFANSSON, S.O. (2008) The effect of temperature and fish size on growth, feed intake, food conversion efficiency and stomach evacuation rate of Atlantic salmon post-smolts. *Aquaculture* **283**, 36–

42.

HE, E. & WURTSBAUGH, W. A. (1993) An Empirical Model of Gastric Evacuation Rates for Fish and an Analysis of Digestion in Piscivorous Brown Trout. *Transactions of the American Fisheries Society* **122**, 717–730.

HE, P. & WARDLE, C.S. (1988) Endurance at intermediate swimming speeds of Atlantic mackerel, Scomber scombrus L., herring, Clupea harengus L., and saithe, Pollachius virens L. *Journal of Fish Biology* **33**, 255–266.

HEDGER, R., HATIN, D., DODSON, J., MARTIN, F., FOURNIER, D., CARON, F. & WHORISKEY, F. (2009) Migration and swimming depth of Atlantic salmon kelts Salmo salar in coastal zone and marine habitats. *Marine Ecology Progress Series* **392**, 179–192.

HEIDELBERG, K., SEBENS, K. & PURCELL, J. (1997) Effects of prey escape behavior and water flow on prey capture by the scleractinian coral, Meandrina meandrites. *Proc 8th Int Coral Reef Symp*, 1081–1086.

HEYWOOD, K.J. (1996) Diel vertical migration of zooplankton in the Northeast Atlantic. *Journal of Plankton Research* **18**, 163–184.

IRIGOIEN, X. (1998) Gut clearance rate constant , temperature and initial gut contents : a review. *Journal of Plankton Research* **20**, 997–1003.

KLEVJER, T. A. & KAARTVEDT, S. (2011) Krill (Meganyctiphanes norvegica) swim faster at night. *Limnology and Oceanography* **56**, 765–774.

LEIS, J. & CARSON-EWART, B. (2000) Behaviour of pelagic larvae of four coral-reef fish species in the ocean and an atoll lagoon. *Coral Reefs* **19**, 247–257.

LINDSTRÖM, M. & FORTELIUS, W. (2001) Swimming behaviour in Monoporeia affinis (Crustacea: Amphipoda) - Dependence on temperature and population density. *Journal of Experimental Marine Biology and Ecology* **256**, 73–83.

VAN DER LINGEN, C.D. (1995) Respiration rate of adult pilchard Sardinops sagax in relation to temperature, voluntary swimming speed and feeding behaviour. *Marine Ecology Progress Series* **129**, 41–54.

LOUGHMAN, Z.J., SKALICAN, K.T. & TAYLOR, N.D. (2013) Habitat selection and movement of Cambarus chasmodactylus (Decapoda:Cambaridae) assessed via radio telemetry. *Freshwater Science* **32**, 1288–1297.

LUCKENBACH, M.W. & ORTH, R.J. (1992) Swimming velocities and behavior of blue crab (Callinectes sapidus Rathbun) megalopae in still and flowing water. *Estuaries* **15**, 186–192.

MCGAW, I.J. & CURTIS, D.L. (2013) A review of gastric processing in decapod crustaceans. *Journal of Comparative Physiology B: Biochemical, Systemic, and Environmental Physiology* **183**, 443–465.

MONTGOMERY, J. & TARGETT, T. (1992) The nutritional role of seagrass in the diet of the omnivorous pinfish Lagodon rhomboides(L.). *Journal of experimental marine biology and ecology* **158**, 37–57.

MURTAUGH, P.A. (1984) Variable gut residence time: Problems in inferring feeding rate from stomach fullness of a mysid crustacean. *CAN. J. FISH. AQUAT. SCI.* **41**, 1287–1293.

NIKOLOPOULOU, D., MOUTOU, K. A, FOUNTOULAKI, E., VENOU, B., ADAMIDOU, S. & ALEXIS, M.N. (2011) Patterns of gastric evacuation, digesta characteristics and pH changes along the gastrointestinal tract of gilthead sea bream (Sparus aurata L.) and European sea bass (Dicentrarchus labrax L.). *Comparative biochemistry and physiology. Part A, Molecular & integrative physiology* **158**, 406–414. Elsevier Inc.

OBER, G.T., THORNBER, C., GREAR, J. & KOLBE, J.J. (2016) Ecological differences influence the thermal sensitivity of swimming performance in two co-occurring mysid shrimp species with climate change implications. *Journal of Thermal Biology*.

ODUM, W.E. (1970) Utilization of the direct grazing and plant detritus food chains by the striped muller Mugil cephalus. In

Marine Food Chains (ed J.H. STEELE), pp. 222–240University. Berkeley and Los Angeles.

ONSRUD, M.S.R. & KAARTVEDT, S. (1998) Diel vertical migration of the krill MEganyctiphanes norvegica in relation to physical environment, food and predators. *Marine Ecology Progress Series* **171**, 209–219.

PÉREZ-CASANOVA, J.C., LALL, S.P. & GAMPERL, A. K. (2009) Effect of feed composition and temperature on food consumption, growth and gastric evacuation of juvenile Atlantic cod (Gadus morhua L.) and haddock (Melanogrammus aeglefinus L.). *Aquaculture* **294**, 228–235. Elsevier B.V.

PERISSINOTTO, R. & PAKHOMOV, E. A. (1996) Gut evacuation rates and pigment destruction in the Antarctic krill Euphausia superba. *Marine Biology* **125**, 47–54.

RANDALL, J.E. (1961) A Contribution to the Biology of the Convict Surgeonfish of the Hawaiian Islands , Acanthurus triostegus sandoicensis. *Pacific Science* **15**, 215–272.

SEYHAN, K., BASCINAR, N.S. & DUZGUNES, E. (1998) Some Observations on Gastric Emptying in the Black Sea Whiting, Merlangius merlangus euxinus.(N. 1840) Fed on Natural Prey. *Turkish Journal of Zoology* **22**, 251–257.

STEINHAUSEN, M.F. (2005) Swimming Costs of Fish. University of Copenhagen.

STENTIFORD, G.D., NEIL, D.M., ATKINSON, R.J.A. & BAILEY, N. (2000) An analysis of swimming performance in the Norway lobster, Nephrops norvegicus L. infected by a parasitic dinoflagellate of the genus Hematodinium. *Journal of Experimental Marine Biology and Ecology* **247**, 169–181.

SUNDSTRÖM, L. & GRUBER, S. (1998) Using speed-sensing transmitters to construct a bioenergetics model for subadult lemon sharks, Negaprion brevirostris (Poey), in the field. *Advances in Invertebrates and Fish Telemetry* **371/372**, 241–247.

SZYPULA, J. & ZALACHOWSKI, W. (1984) Duration of food evacuation in herring, Clupea harengus L., and sprat, Sprattus sprattus (L.). *Acta ichthyologica et piscatoria* **14**, 93–104.

TEMMING, A., BOHLET, B., SKAGEN, D.W. & KNUDSEN, F.R. (2002) Gastric evacuation in mackerel: the effects of meal size, prey type and temperature. *Journal of Fish Biology* **61**, 50–70.

VAGNER, M., LEFRANÇOIS, C., FERRARI, R.S., SATTA, A. & DOMENICI, P. (2008) The effect of acute hypoxia on swimming stamina at optimal swimming speed in flathead grey mullet Mugil cephalus. *Marine Biology* **155**, 183–190.

WATANABE, Y.Y., SATO, K., WATANUKI, Y., TAKAHASHI, A., MITANI, Y., AMANO, M., AOKI, K., NARAZAKI, T., IWATA, T., MINAMIKAWA, S. & MIYAZAKI, N. (2011) Scaling of swim speed in breath-hold divers. *The Journal of animal ecology* **80**, 57–68.

WETHERBEE, B. & GRUBER, S. (1990) The Effects of Ration Level on food retention time in juvenile lemon sharks, Negaprion brevirostris. *Environmental biology of fishes* **29**, 59–65.

WILLOUGHBY, L.G. & EARNSHAW, R. (1982) Gut passage times in Gammarus pulex (Crustacea, Amphipoda) and aspects of summer feeding in a stony stream. *Hydrobiologia* **97**, 105–117.

YAMANAKA, K. LYNNE & RICHARDS, L.J. (1993) Movements of transplanted lingcod, Ophiodon elongatus, determined by ultrasonic telemetry. *Fishery Bulletin* **587**, 582–587.

YAMAZAKI, H. & SQUIRES, K. (1996) Comparison of oceanic turbulence and copepod swimming. *Marine Ecology Progress Series* **144**, 299–301.

3.2 – References used in Table S2

ABDELRHMAN, K.F.A., BACCI, G., MARRAS, B., NISTRI, A., SCHINTU, M., UGOLINI, A. & MENGONI, A.

(2016) Exploring the bacterial gut microbiota of supralittoral talitrid amphipods. *Research in Microbiology* **168**, 1–11. Elsevier Masson SAS.

AIRES, T., SERRÃO, E.A., KENDRICK, G., DUARTE, C.M. & ARNAUD-HAOND, S. (2013) Invasion Is a Community Affair: Clandestine Followers in the Bacterial Community Associated to Green Algae, Caulerpa racemosa, Track the Invasion Source. *PLoS ONE* **8**.

ANOOP, A. & ANTUNES, A. (2015) Pyrosequencing Characterization of the Microbiota from Atlantic Intertidal Marine Sponges Reveals High Microbial Diversity and the Lack of Co-Occurrence Patterns. *Plos One* **10**, e0127455.

ARELLANO, S.M., LEE, O.O., LAFI, F.F., YANG, J., WANG, Y., YOUNG, C.M. & QIAN, P.Y. (2013) Deep Sequencing of Myxilla (Ectyomyxilla) methanophila, an Epibiotic Sponge on Cold-Seep Tubeworms, Reveals Methylotrophic, Thiotrophic, and Putative Hydrocarbon-Degrading Microbial Associations. *Microbial Ecology* **65**, 450–461.

BALCÁZAR, J.L., LEE, N.M., PINTADO, J. & PLANAS, M. (2010) Phylogenetic characterization and in situ detection of bacterial communities associated with seahorses (Hippocampus guttulatus) in captivity. *Systematic and applied microbiology* **33**, 71–77.

BENGTSSON, M.M., SJØTUN, K., LANZÉN, A. & ØVREÅS, L. (2012) Bacterial diversity in relation to secondary production and succession on surfaces of the kelp Laminaria hyperborea. *Isme J* **6**, 2188–2198.

BIK, E.M., COSTELLO, E.K., SWITZER, A.D., CALLAHAN, B.J., HOLMES, S.P., WELLS, R.S., CARLIN, K.P., JENSEN, E.D., VENN-WATSON, S. & RELMAN, D.A. (2016) Marine mammals harbor unique microbiotas shaped by and yet distinct from the sea. *Nature Communications* **7**, 10516. Nature Publishing Group.

BRODIE, J., WILLIAMSON, C., BARKER, G.L., WALKER, R.H., BRISCOE, A. & YALLOP, M. (2016) Characterising the microbiome of Corallina officinalis, a dominant calcified intertidal red alga. *FEMS Microbiology Ecology* **92**, 1–12.

BURKE, C., THOMAS, T., LEWIS, M., STEINBERG, P. & KJELLEBERG, S. (2011) Composition, uniqueness and variability of the epiphytic bacterial community of the green alga Ulva australis. *The ISME journal* **5**, 590–600. Nature Publishing Group.

CARDA-DIÉGUEZ, M., MIRA, A. & FOUZ, B. (2014) Pyrosequencing survey of intestinal microbiota diversity in cultured sea bass (Dicentrarchus labrax) fed functional diets. *FEMS Microbiology Ecology* **87**, 451–459.

DE CASTRO, A.P., ARAÚJO, S.D., REIS, A.M.M., POMPEU, M., HATAY, M., DE MOURA, R.L., FRANCINI-FILHO, R.B., THOMPSON, F.L. & KRÜGER, R.H. (2013) Bacterial communities associated with three Brazilian endemic reef corals (Mussismilia spp.) in a coastal reef of the Abrolhos shelf. *Continental Shelf Research* **70**, 135–139. Elsevier.

CUVELIER, M.L., BLAKE, E., MULHERON, R., MCCARTHY, P.J., BLACKWELDER, P., THURBER, R.L.V. &

LOPEZ, J. V. (2014) Two distinct microbial communities revealed in the sponge cinachyrella. *Frontiers in Microbiology* **5**, 1–12.

DUPONT, S., CORRE, E., LI, Y., VACELET, J. & BOURGUET-KONDRACKI, M.-L. (2013) First insights into the microbiome of a carnivorous sponge. *FEMS microbiology ecology*.

DURAND, L., ZBINDEN, M., CUEFF-GAUCHARD, V., DUPERRON, S., ROUSSEL, E.G., SHILLITO, B. & CAMBON-BONAVITA, M.-A. (2009) Microbial diversity associated with the hydrothermal shrimp Rimicaris exoculata gut and occurrence of a resident microbial community. *FEMS Microbiology Ecology* **71**, 291–303.

ERWIN, P.M., OLSON, J.B. & THACKER, R.W. (2011) Phylogenetic diversity, host-specificity and community profiling of sponge-associated bacteria in the northern Gulf of Mexico. *PloS one* **6**, e26806.

ERWIN, P.M., PINEDA, M.C., WEBSTER, N., TURON, X. & LÓPEZ-LEGENTIL, S. (2014) Down under the tunic: bacterial biodiversity hotspots and widespread ammonia-oxidizing archaea in coral reef ascidians. *The ISME journal* **8**, 575–588.

FERNANDES, N., STEINBERG, P., RUSCH, D., KJELLEBERG, S. & THOMAS, T. (2012) Community Structure and Functional Gene Profile of Bacteria on Healthy and Diseased Thalli of the Red Seaweed Delisea pulchra. *PLoS ONE* **7**.

FERNANDO, S.C., WANG, J., SPARLING, K., GARCIA, G.D., FRANCINI-FILHO, R.B., DE MOURA, R.L., PARANHOS, R., THOMPSON, F.L. & THOMPSON, J.R. (2014) Microbiota of the Major South Atlantic Reef Building Coral Mussismilia. *Microbial Ecology* **69**, 267–280.

FIDOPIASTIS, P.M., BEZDEK, D.J., HORN, M.H. & KANDEL, J.S. (2006) Characterizing the resident, fermentative microbial consortium in the hindgut of the temperate-zone herbivorous fish, Hermosilla azurea (Teleostei: Kyphosidae). *Marine Biology* **148**, 631–642.

FRIAS-LOPEZ, J., ZERKLE, A.L., BONHEYO, G.T. & FOUKE, B.W. (2002) Partitioning of bacterial communities between seawater and healthy, black band diseased, and dead coral surfaces. *Applied and environmental microbiology* **68**, 2214–2228.

GAO, F., LI, F., TAN, J., YAN, J. & SUN, H. (2014) Bacterial community composition in the gut content and ambient sediment of sea cucumber Apostichopus japonicus revealed by 16S rRNA gene pyrosequencing. *PLoS ONE* **9**, 1–10.

GERDTS, G., BRANDT, P., KREISEL, K., BOERSMA, M., SCHOO, K.L. & WICHELS, A. (2013) The microbiome of North Sea copepods. *Helgoland Marine Research* **67**, 757–773.

GIVENS, C.E., RANSOM, B., BANO, N. & HOLLIBAUGH, J.T. (2015) Comparison of the gut microbiomes of 12 bony fish and 3 shark species. *Marine Ecology Progress Series* **518**, 209–223.

GODOY-VITORINO, F., RODRIGUEZ-HILARIO, A., ALVES, A.L., GON??ALVES, F., CABRERA-COLON, B., MESQUITA, C.S., SOARES-CASTRO, P., FERREIRA, M., MAR??ALO, A., VINGADA, J., EIRA, C. & SANTOS, P.M. (2016) The microbiome of a striped dolphin (Stenella coeruleoalba) stranded in

Portugal. *Research in Microbiology* **168**, 85–93.

GULMANN, L.K. (2004) Gut-associated microbial symbionts of the marsh fiddler crab, Uca pugnax. Woods Hole Ovceanographic Institution.

HERNANDEZ-ZULUETA, J., ARAYA, R., VARGAS-PONCE, O., DIAZ-PEREZ, L., RODRIGUEZ-TRONCOSO, A.P., CEH, J., RIOS-JARA, E. & RODRIGUEZ-ZARAGOZA, F.A. (2016) First deep screening of bacterial assemblages associated with corals of the Tropical Eastern Pacific. *FEMS Microbiology Ecology* **92**, 1–12.

HOLBEN, W.E., WILLIAMS, P., GILBERT, M. A, SAARINEN, M., SÄRKILAHTI, L.K. & APAJALAHTI, J.H. A (2002) Phylogenetic analysis of intestinal microflora indicates a novel Mycoplasma phylotype in farmed and wild salmon. *Microbial ecology* **44**, 175–185.

IEHATA, S., VALENZUELA, F. & RIQUELME, C. (2013) Analysis of bacterial community and bacterial nutritional enzyme activity associated with the digestive tract of wild Chilean octopus (Octopus mimus Gould, 1852). *Aquaculture Research*, 861–873.

JACKSON, S. A, KENNEDY, J., MORRISSEY, J.P., O'GARA, F. & DOBSON, A.D.W. (2012) Pyrosequencing reveals diverse and distinct sponge-specific microbial communities in sponges from a single geographical location in Irish waters. *Microbial ecology* **64**, 105–116.

KARLIŃSKA-BATRES, K. & WÖRHEIDE, G. (2013) Phylogenetic Diversity and Community Structure of the Symbionts Associated with the Coralline Sponge Astrosclera willeyana of the Great Barrier Reef. *Microbial Ecology* **65**, 740–752.

KARLINSKA-BATRES, K. & WÖRHEIDE, G. (2015) Spatial variability of microbial communities of the coralline demosponge Astrosclera willeyana across the Indo-Pacific. *Aquatic Microbial Ecology* **74**, 143–156.

KELLOGG, C. A, LISLE, J.T. & GALKIEWICZ, J.P. (2009) Culture-independent characterization of bacterial communities associated with the cold-water coral Lophelia pertusa in the northeastern Gulf of Mexico. *Applied and environmental microbiology* **75**, 2294–2303.

KIM, D.H., BRUNT, J. & AUSTIN, B. (2007) Microbial diversity of intestinal contents and mucus in rainbow trout (Oncorhynchus mykiss). *Journal of Applied Microbiology* **102**, 1654–1664.

KING, G.M., JUDD, C., KUSKE, C.R. & SMITH, C. (2012) Analysis of stomach and gut microbiomes of the eastern oyster (Crassostrea virginica) from coastal Louisiana, USA. *PloS one* **7**, e51475. Public Library Science.

KORMAS, K. A, MEZITI, A., MENTE, E. & FRENTZOS, A. (2014) Dietary differences are reflected on the gut prokaryotic community structure of wild and commercially reared sea bream (Sparus aurata). *MicrobiologyOpen*.

LAU, W.W.Y., JUMARS, P. A. & ARMBRUST, E.V. (2002) Genetic Diversity of Attached Bacteria in the Hindgut of the Deposit-Feeding Shrimp Neotrypaea (formerly Callianassa) californiensis (Decapoda: Thalassinidae). *Microbial Ecology* **43**, 455–466.

LEE, O.O., WANG, Y., YANG, J., LAFI, F.F., AL-SUWAILEM, A. & QIAN, P.-Y. (2011) Pyrosequencing reveals highly diverse and species-specific microbial communities in sponges from the Red Sea. *The ISME journal* **5**, 650–664. Nature Publishing Group.

LI, J., CHEN, Q., LONG, L., DONG, J., YANG, J. & ZHANG, S. (2014) Bacterial dynamics within the mucus, tissue and skeleton of the coral Porites lutea during different seasons. *Scientific Reports*, 1–8.

LI, K., GUAN, W., WEI, G., LIU, B., XU, J., ZHAO, L. & ZHANG, Y. (2007) Phylogenetic analysis of intestinal bacteria in the Chinese mitten crab (Eriocheir sinensis). *Journal of applied microbiology* **103**, 675–682.

LI, S., SUN, L., WU, H., HU, Z., LIU, W., LI, Y. & WEN, X. (2012) The intestinal microbial diversity in mud crab (Scylla paramamosain) as determined by PCR-DGGE and clone library analysis. *Journal of applied microbiology* **113**, 1341–1351.

LINS-DE-BARROS, M.M., VIEIRA, R.P., CARDOSO, A.M., MONTEIRO, V. A, TURQUE, A.S., SILVEIRA, C.B., ALBANO, R.M., CLEMENTINO, M.M. & MARTINS, O.B. (2010) Archaea, Bacteria, and algal plastids associated with the reef-building corals Siderastrea stellata and Mussismilia hispida from Búzios, South Atlantic Ocean, Brazil. *Microbial ecology* **59**, 523–532.

LONGFORD, S.R., TUJULA, N.A., CROCETTI, G.R., HOLMES, A.J., HOLMSTRÖM, C., KJELLEBERG, S., STEINBERG, P.D. & TAYLOR, M.W. (2007) Comparisons of diversity of bacterial communities associated with three sessile marine eukaryotes. *Aquatic Microbial Ecology* **48**, 217–229.

LÓPEZ-LEGENTIL, S., TURON, X. & ERWIN, P.M. (2016) Feeding cessation alters host morphology and bacterial communities in the ascidian Pseudodistoma crucigaster. *Frontiers in Zoology* **13**, 2. Frontiers in Zoology.

LUTER, H.M., WIDDER, S., BOTTÉ, E.S., ABDUL WAHAB, M., WHALAN, S., MOITINHO-SILVA, L., THOMAS, T. & WEBSTER, N.S. (2015) Biogeographic variation in the microbiome of the ecologically important sponge, Carteriospongia foliascens. *PeerJ* **3**, e1435.

MANCUSO, F.P., D'HONDT, S., WILLEMS, A., AIROLDI, L. & DE CLERCK, O. (2016) Diversity and temporal dynamics of the epiphytic bacterial communities associated with the canopy-forming seaweed Cystoseira compressa (Esper) Gerloff and Nizamuddin. *Frontiers in Microbiology* **7**, 1–11.

MEZITI, A., RAMETTE, A., MENTE, E. & KORMAS, K.A. (2010) Temporal shifts of the Norway lobster (Nephrops norvegicus) gut bacterial communities. *FEMS microbiology ecology* **74**, 472–484.

MIRANDA, L.N., HUTCHISON, K., GROSSMAN, A.R. & BRAWLEY, S.H. (2013) Diversity and Abundance of the Bacterial Community of the Red Macroalga Porphyra umbilicalis: Did Bacterial Farmers Produce Macroalgae? *PLoS ONE* **8**.

MIYAKE, S., NGUGI, D.K. & STINGL, U. (2015) Diet strongly influences the gut microbiota of surgeonfishes. *Molecular Ecology* **24**, 656–672.

MOISANDER, P.H., SEXTON, A.D. & DALEY, M.C. (2015) Stable associations masked by temporal variability in the marine copepod microbiome. *PLoS ONE* **10**, 1–17.

NELSON, L., BLAIR, B., MURDOCK, C., MEADE, M., WATTS, S. & LAWRENCE, A.L. (2010) Molecular Analysis of Gut Microflora in Captive-Raised Sea Urchins (Lytechinus variegatus). *Journal of the World Aquaculture Society* **41**, 807–815.

O'CONNOR-SÁNCHEZ, A., RIVERA-DOMÍNGUEZ, A.J., DE LOS SANTOS-BRIONES, C., LÓPEZ-AGUIAR, L.K., PEÑA-RAMÍREZ, Y.J. & PRIETO-DAVO, A. (2014) Acidobacteria appear to dominate the microbiome of two sympatric Caribbean Sponges and one Zoanthid. *Biological Research* **47**, 67.

PARRIS, D.J., BROOKER, R.M., MORGAN, M.A., DIXSON, D.L. & STEWART, F.J. (2016) Whole gut microbiome composition of damselfish and cardinalfish before and after reef settlement. *PeerJ* **4**, e2412.

POLÓNIA, A.R.M., CLEARY, D.F.R., DUARTE, L.N., DE VOOGD, N.J. & GOMES, N.C.M. (2014) Composition of Archaea in Seawater, Sediment, and Sponges in the Kepulauan Seribu Reef System, Indonesia. *Microbial Ecology*, 1–15.

PRICE, J.T. (2016) Characterization of the Juvenile Green Turtle (Chelonia mydas) Microbiome Associated with the Ontogenic Shift from Pelagic Habitats to Inshore Resident Areas.

RADWAN, M., HANORA, A., ZAN, J., MOHAMED, N.M., ABO-ELMATTY, D.M., ABOU-EL-ELA, S.H. & HILL, R.T. (2010) Bacterial community analyses of two Red Sea sponges. *Marine biotechnology (New York, N.Y.)* **12**, 350–360.

RANSON, B.L. (2008) Intestinal microbial community composition of six actinopterygii fish species in the southeastern united states. University of Georgia.

REVEILLAUD, J., MAIGNIEN, L., EREN, M. A, HUBER, J. A, APPRILL, A., SOGIN, M.L. & VANREUSEL, A. (2014) Host-specificity among abundant and rare taxa in the sponge microbiome. *The ISME journal* **8**, 1198–1209.

ROHWER, F., SEGURITAN, V., AZAM, F. & KNOWLTON, N. (2002) Diversity and distribution of coral-associated bacteria. *Marine Ecology Progress Series* **243**, 1–10.

RUNGRASSAMEE, W., KLANCHUI, A., CHAIYAPECHARA, S., MAIBUNKAEW, S., TANGPHATSORNRUANG, S., JIRAVANICHPAISAL, P. & KAROONUTHAISIRI, N. (2013) Bacterial population in intestines of the black tiger shrimp (Penaeus monodon) under different growth stages. *PloS one* **8**, e60802.

RUNGRASSAMEE, W., KLANCHUI, A., MAIBUNKAEW, S., CHAIYAPECHARA, S., JIRAVANICHPAISAL, P. & KAROONUTHAISIRI, N. (2014) Characterization of intestinal bacteria in wild and domesticated adult black tiger shrimp (Penaeus monodon). *PLoS ONE* **9**.

SCHMITT, S., TSAI, P., BELL, J., FROMONT, J., ILAN, M., LINDQUIST, N., PEREZ, T., RODRIGO, A., SCHUPP, P.J., VACELET, J., WEBSTER, N., HENTSCHEL, U. & TAYLOR, M.W. (2012) Assessing the complex sponge microbiota: core, variable and species-specific bacterial communities in marine sponges. Nature Publishing Group. *The ISME Journal*. .

SEKAR, R., KACZMARSKY, L.T. & RICHARDSON, L.L. (2008) Microbial community composition of black band disease on the coral host Siderastrea siderea from three regions of the wider Caribbean. *Marine Ecology Progress Series* **362**, 85–98.

SEKAR, R., MILLS, D.K., REMILY, E.R., VOSS, J.D. & RICHARDSON, L.L. (2006) Microbial communities in the surface mucopolysaccharide layer and the black band microbial mat of black band-diseased Siderastrea siderea. *Applied and Environmental Microbiology* **72**, 5963–5973.

SHIINA, A., ITOI, S., WASHIO, S. & SUGITA, H. (2006) Molecular identification of intestinal microflora in Takifugu niphobles. *Comparative biochemistry and physiology. Part D, Genomics & proteomics* **1**, 128–132.

SHOEMAKER, K.M. & MOISANDER, P.H. (2014) Microbial diversity associated with copepods in the North Atlantic subtropical gyre. *FEMS Microbiology Ecology*, 1–11.

SKEA, G.L., MOUNTFORT, D.O. & CLEMENTS, K.D. (2007) Contrasting digestive strategies in four New Zealand herbivorous fishes as reflected by carbohydrase activity profiles. *Comparative biochemistry and physiology. Part A, Molecular & integrative physiology* **146**, 63–70.

SMRIGA, S., SANDIN, S.A. & AZAM, F. (2010) Abundance, diversity, and activity of microbial assemblages associated with coral reef fish guts and feces. *FEMS Microbiology Ecology* **73**, 31–42.

SONG, W., LI, L., HUANG, H., JIANG, K., ZHANG, F., CHEN, X., ZHAO, M. & MA, L. (2016) The Gut Microbial Community of Antarctic Fish Detected by 16S rRNA Gene Sequence Analysis. *BioMed Research International* **2016**.

SOVERINI, M., QUERCIA, S., BIANCANI, B., FURLATI, S., TURRONI, S., BIAGI, E., CONSOLANDI, C., PEANO, C., SEVERGNINI, M., RAMPELLI, S., BRIGIDI, P. & CANDELA, M. (2016) The bottlenose dolphin (*Tursiops truncatus*) faecal microbiota. *FEMS Microbiology Ecology* **92**, fiw055.

STAR, B., HAVERKAMP, T.H. A, JENTOFT, S. & JAKOBSEN, K.S. (2013) Next generation sequencing shows high variation of the intestinal microbial species composition in Atlantic cod caught at a single location. *BMC microbiology* **13**, 248.

SULLAM, K.E., ESSINGER, S.D., LOZUPONE, C.A., O'CONNOR, M.P., ROSEN, G.L., KNIGHT, R., KILHAM, S.S. & RUSSELL, J.A. (2012) Environmental and ecological factors that shape the gut bacterial communities of fish: a meta-analysis. *Molecular Ecology* **21**, 3363–3378.

SUN, W., ZHANG, F., HE, L. & LI, Z. (2014) Pyrosequencing Reveals Diverse Microbial Community Associated with the Zoanthid Palythoa australiae from the South China Sea. *Microbial Ecology* **67**, 942–950.

SUNAGAWA, S., DESANTIS, T.Z., PICENO, Y.M., BRODIE, E.L., DESALVO, M.K., VOOLSTRA, C.R., WEIL, E., ANDERSEN, G.L. & MEDINA, M. (2009) Bacterial diversity and White Plague Disease-associated community changes in the Caribbean coral Montastraea faveolata. *The ISME journal* **3**, 512–521.

SUNAGAWA, S., WOODLEY, C.M. & MEDINA, M. (2010) Threatened corals provide underexplored microbial habitats. *PLoS ONE* **5**, 1–7.

TANAKA, R., OOTSUBO, M., SAWABE, T., EZURA, Y. & TAJIMA, K. (2004) Biodiversity and in situ abundance of gut microflora of abalone (Haliotis discus hannai) determined by culture-independent techniques. *Aquaculture* **241**, 453–463.

TARNECKI, A.M., PATTERSON, W.F. & ARIAS, C.R. (2016) Microbiota of wild-caught Red Snapper Lutjanus campechanus. *BMC Microbiology* **16**, 245. BMC Microbiology.

THOMAS, T., MOITINHO-SILVA, L., LURGI, M., BJÖRK, J.R., EASSON, C., ASTUDILLO-GARCÍA, C., ET AL. (2016) Diversity, structure and convergent evolution of the global sponge microbiome. *Nature communications* **7**, 11870.

WARD, N.L., STEVEN, B., PENN, K., METHÉ, B. A & DETRICH, W.H. (2009) Characterization of the intestinal microbiota of two Antarctic notothenioid fish species. *Extremophiles : life under extreme conditions* **13**, 679–685.

WEBSTER, N.S., TAYLOR, M.W., BEHNAM, F., LÜCKER, S., RATTEI, T., WHALAN, S., HORN, M. & WAGNER, M. (2010) Deep sequencing reveals exceptional diversity and modes of transmission for bacterial sponge symbionts. *Environmental microbiology* **12**, 2070–2082.

WU, S., GAO, T., ZHENG, Y., WANG, W., CHENG, Y. & WANG, G. (2010) Microbial diversity of intestinal contents and mucus in yellow catfish (Pelteobagrus fulvidraco). *Aquaculture* **303**, 1–7. Elsevier B.V.

XING, M., HOU, Z., YUAN, J., LIU, Y., QU, Y. & LIU, B. (2013) Taxonomic and functional metagenomic profiling of gastrointestinal tract microbiome of the farmed adult turbot (Scophthalmus maximus). *FEMS Microbiology Ecology* **86**, 432–443.

ZOZAYA-VALDES, E., EGAN, S. & THOMAS, T. (2015) A comprehensive analysis of the microbial communities of healthy and diseased marine macroalgae and the detection of known and potential bacterial pathogens. *Frontiers in Microbiology* **6**, 1–9.

ZOZAYA-VALDÉS, E., ROTH-SCHULZE, A.J. & THOMAS, T. (2016) Effects of temperature stress and aquarium conditions on the red macroalga Delisea pulchra and its associated microbial community. *Frontiers in Microbiology* **7**, 1–10.

3.3 – References used in Table S3

AL-HARBI, A.H. & UDDIN, M.N. (2004) Seasonal variation in the intestinal bacterial flora of hybrid tilapia (Oreochromis niloticus x Oreochromis aureus) cultured in earthen ponds in Saudi Arabia. *Aquaculture* **229**, 37–44.

FIDOPIASTIS, P.M., BEZDEK, D.J., HORN, M.H. & KANDEL, J.S. (2006) Characterizing the resident, fermentative microbial consortium in the hindgut of the temperate-zone herbivorous fish, Hermosilla azurea (Teleostei: Kyphosidae). *Marine Biology* **148**, 631–642.

HOLBEN, W.E., WILLIAMS, P., GILBERT, M. A, SAARINEN, M., SÄRKILAHTI, L.K. & APAJALAHTI, J.H. A (2002) Phylogenetic

analysis of intestinal microflora indicates a novel Mycoplasma phylotype in farmed and wild salmon. *Microbial ecology* **44**, 175–185.

KIM, D.H., BRUNT, J. & AUSTIN, B. (2007) Microbial diversity of intestinal contents and mucus in rainbow trout (Oncorhynchus mykiss). *Journal of Applied Microbiology* **102**, 1654–1664.

RINGØ, E. (1993) Does chromic oxide (Cr-2O-3) affect faecal lipid and intestinal bacterial flora in Arctic charr, Salvelinus alpinus (L.)? *Aquaculture and Fisheries Management* **24**, 767–776.

RINGØ, E., SPERSTAD, S., MYKLEBUST, R., REFSTIE, S. & KROGDAHL, Å. (2006) Characterisation of the microbiota associated with intestine of Atlantic cod (Gadus morhua L.). *Aquaculture* **261**, 829–841.

RINGØ, E. & STRØM, E. (1994) Microflora of Arctic charr, Salvelinus alpinus (L.): gastrointestinal microflora of free-living fish and effect of diet and salinity on intestinal microflora. *Aquaculture Research* **25**, 623–629.

ROMERO, J. & NAVARRETE, P. (2006) 16S rDNA-based analysis of dominant bacterial populations associated with early life stages of coho salmon (Oncorhynchus kisutch). *Microbial ecology* **51**, 422–430.

SHIINA, A., ITOI, S., WASHIO, S. & SUGITA, H. (2006) Molecular identification of intestinal microflora in Takifugu niphobles. *Comparative biochemistry and physiology. Part D, Genomics & proteomics* **1**, 128–132.

SMRIGA, S., SANDIN, S.A. & AZAM, F. (2010) Abundance, diversity, and activity of microbial assemblages associated with coral reef fish guts and feces. *FEMS Microbiology Ecology* **73**, 31–42.

SPANGGAARD, B., HUBER, I., NIELSEN, J., NIELSEN, T., APPEL, K.. & GRAM, L. (2000) The microflora of rainbow trout intestine: a comparison of traditional and molecular identification. *Aquaculture* **182**, 1–15.

SUGITA, H., KUROSAKI, M., OKAMURA, T. & YAMAMOTO, S. (2005) The culturability of intestinal bacteria of Japanese coastal fish. *Fisheries Science* **71**, 956–958.

SUN, Y., YANG, H., LING, Z., CHANG, J. & YE, J. (2009) Gut microbiota of fast and slow growing grouper Epinephelus coioides. *African Journal of Microbiology Research* **3**, 713–720.

SVANEVIK, C. & LUNESTAD, B. (2011) Characterisation of the microbiota of Atlantic mackerel (Scomber scombrus). *International Journal of Food Microbiology* **151**, 164–170. Elsevier B.V.

YANO, Y., NAKAYAMA, A. & YOSHIDA, K. (1995) Population sizes and growth pressure responses of intestinal microfloras of deep-sea fish retrieved from the abyssal zone. *Applied and Environmental Microbiology* **61**, 4480–4483.
